# Emotion Processing and Its Relationship to Social Functioning and Symptoms in Psychotic Disorder: A Systematic Review and Meta-analysis

**DOI:** 10.1093/schbul/sbae167

**Published:** 2024-09-04

**Authors:** Sean Murrihy, Kate Filia, Sue Cotton, Lisa Phillips, Sarah Youn, Anuradhi Jayasinghe, Anna Wrobel, Eslam M Bastawy, Kelly Allott, Amity Watson

**Affiliations:** Melbourne School of Psychological Sciences, University of Melbourne, Melbourne 3052, Australia; Orygen, Melbourne 3052, Australia; Centre for Youth Mental Health, University of Melbourne, Melbourne 3052, Australia; Orygen, Melbourne 3052, Australia; Centre for Youth Mental Health, University of Melbourne, Melbourne 3052, Australia; Orygen, Melbourne 3052, Australia; Centre for Youth Mental Health, University of Melbourne, Melbourne 3052, Australia; Melbourne School of Psychological Sciences, University of Melbourne, Melbourne 3052, Australia; Orygen, Melbourne 3052, Australia; School of Psychology, Deakin University, Geelong 3220, Australia; Orygen, Melbourne 3052, Australia; School of Psychology, Deakin University, Geelong 3220, Australia; Orygen, Melbourne 3052, Australia; IMPACT, The Institute for Mental and Physical Health and Clinical Translation, Deakin University, Geelong 3220, Australia; IMPACT, The Institute for Mental and Physical Health and Clinical Translation, Deakin University, Geelong 3220, Australia; Faculty of Science, Ain Shams University, Cairo 11566, Egypt; Orygen, Melbourne 3052, Australia; Centre for Youth Mental Health, University of Melbourne, Melbourne 3052, Australia; Orygen, Melbourne 3052, Australia; Centre for Youth Mental Health, University of Melbourne, Melbourne 3052, Australia

**Keywords:** social cognition, positive symptoms, negative symptoms, disorganization symptoms, depressive symptoms, first episode psychosis, schizophrenia

## Abstract

**Background:**

Emotion processing (EP) is impaired in individuals with psychosis and associated with social functioning; however, it is unclear how symptoms fit into this relationship. The aim of this systematic review and meta-analysis was to examine interrelationships between EP, symptoms, and social functioning, test whether different symptom domains mediate the relationship between EP and social functioning, and examine the moderating effects of illness stage and EP task type.

**Study Design:**

MEDLINE, Embase, and PsycINFO databases were searched for studies that included individuals with psychosis and reported correlations between EP, symptom domains (positive, negative, depressive, and disorganization), and social functioning. Random effects meta-analyses determined the strength of correlations, and subgroup analyses included illness stage and EP task type (lower- vs higher-level processing). Meta-analytic structural equation models tested whether symptom domains mediated the relationship between EP and social functioning.

**Results:**

There was a small relationship (*r* = .18) between EP and social functioning. Positive, negative, and disorganization symptoms mediated this relationship, although indirect effects were small. Higher-level EP tasks were more strongly associated with negative symptoms than lower-level tasks. Relationships between EP and both social functioning and positive symptoms were smaller in the first episode of psychosis than in established illness.

**Conclusions:**

The mediating relationship suggests that EP not only influences social dysfunction directly but contributes to negative and disorganization symptoms, which in turn impair social functioning. This pathway suggests that targeting negative and disorganization symptoms may ultimately improve social outcomes for individuals with psychosis. Future research, particularly in early psychosis, is needed to determine other factors impacting these interrelationships.

## Introduction

Social cognitive impairments are a trait-like feature of psychotic disorders that typically emerge before psychosis onset and persist through all stages of illness.^1,2^ Emotion processing (EP) is one domain of social cognition known to be impaired in individuals with psychotic disorders.^3–5^ EP broadly refers to the ability to perceive and use emotions and can be divided into two components: lower-level processing which involves the perception and identification of emotional displays in social interactions, and higher-level processing which allows one to understand and manage one’s own and others’ emotions effectively.^6,7^ Social cognitive abilities, including EP, represent a promising treatment target, particularly for improving social functioning.^8^

Social functioning refers to an individual’s capacity to both effectively and appropriately interact with others and to function in societally defined roles within the community such as work, activities of daily living, or relationships.^9,10^ Difficulties with EP are associated with poorer social functioning in schizophrenia^9,11,12^ and a recent comprehensive review demonstrated small to moderate relationships between EP and community functioning in non-affective psychosis.^13^ Despite these demonstrated links between EP and social functioning in psychosis, several unresolved questions remain, answers to which may aid the development of interventions to improve social functioning.

First, impairments in EP or other social cognitive domains do not fully explain difficulties in social functioning experienced by those with psychosis, and more complex models including other factors such as symptoms may provide a better account of the relationship between EP and social functioning.^13,14^ Some researchers have suggested that symptoms may represent observable “real-world” behavioral expressions of social cognitive deficits,^15^ that may then have a downstream effect on functioning. For example, difficulties in EP such as recognizing another’s facial expressions, could impair a person’s ability to mirror another’s facial expression, which may manifest as blunted affect. Similarly, misinterpreting another’s expression (ie, as angry or disgusted) could lead to symptoms such as paranoia or depression. These symptoms may then in turn cause difficulties in social interactions, potentially reducing social opportunities such as employment or impacting the quality of relationships.

Several studies have reported a significant indirect relationship between cognition and social functioning that is mediated by symptoms, in particular negative symptoms.^16–18^ While a previous meta-analytic study examined the direct relationships between social cognition and symptoms in schizophrenia, demonstrating small to moderate associations (*r* = −.17 to *r* = −.32) between EP and positive, negative, and disorganization symptoms^19^; it was published more than 10 years ago and did not consider depressive symptoms, which co-occur with high prevalence in psychotic disorders.^20^ Further, no meta-analyses have systematically examined the relationships between EP, symptoms, and social functioning. Testing models where specific symptom domains mediate the relationship between social cognition and social functioning may provide valuable insights into the possible mechanisms underlying the relationship between, and the primacy of, these constructs. Ultimately this could flow through to the development of interventions that more effectively improve social functioning.

Second, it is unclear whether the relationships between EP, symptoms, and social functioning vary over the course of psychotic illness. While EP impairments appear to remain stable throughout the first 5 years of illness,^21,22^ symptom trajectories can be quite variable, with positive symptoms tending to reduce and stabilize over time.^23^ Additionally, different social challenges may be experienced at different stages of illness. For example, first episode psychosis (FEP) typically occurs in adolescence or early adulthood, a period of developmentally important social transitions, such as transitioning from school to further education or the workforce,^24,25^ while older individuals experiencing chronic, long-standing psychotic illnesses may also be managing physical health challenges. Having a better understanding of the relationships between these constructs across all illness stages may be important for developing early interventions to prevent or minimize long-term social dysfunction, as well as tailoring interventions for the later stages of these disorders.

Finally, previous reviews examining relationships between EP and symptoms or social functioning have either only considered lower-level EP abilities,^4,12^ or grouped lower-level and higher-level EP abilities together.^11,13,19^ Factor analytic studies have shown that higher- and lower-level social cognitive processes may differentially relate to symptoms and social functioning.^26,27^ Riedel et al.^27^ demonstrated that “management of emotions” mapped onto a higher-level factor, which was related to community functioning and negative symptoms, whereas “facial emotion recognition” mapped onto a lower-level factor which was not related to negative symptoms or community functioning. Given the wide array of EP tasks used in previous research, examining lower-level and higher-level tasks separately may provide more detailed insights about relationships with specific symptom domains or social functioning.

The aims of this review and meta-analysis were to (1) examine interrelationships between EP, symptoms, and social functioning in psychosis; (2) explore whether these relationships are moderated by stage of the illness; (3) examine effects of task type (ie, level of EP) on the relationships between EP and both symptoms and social functioning; and (4) determine whether symptoms mediate the relationship between EP and social functioning.

## Methods

### Protocol Registration and Standards

This meta-analysis forms part of a larger systematic review pre-registered with the International Prospective Register of Systematic Reviews (PROSPERO, CRD42021283837) examining relationships between social cognition, symptoms, and social functioning across different stages of psychotic disorder. It was conducted in accordance with Preferred Reporting Items for Systemic Reviews and Meta-Analysis (PRISMA) guidelines.^28^ The PRIMSA checklist is available in Table S1.

### Search Strategy

A search was conducted on September 5, 2023, across the databases MEDLINE, Embase, and PsycINFO. The search strategy for the overarching review included keywords related to psychosis (eg, *schizophrenia*, *psychosis*) and social cognition (eg, *EP*, *prosody*) combined with terms related to symptoms (eg, *hallucinations*, *anhedonia*) or social functioning (eg, *social adjustment*), adapted for database differences (Tables S2-S4). Limits were placed on publication date (ie, after 1990), article type (eg, *NOT book chapter*), and language (ie, English). The search was limited to articles published after 1990, as many studies published before this date used non-standardized measures of social cognition and were deemed in an early review to have serious methodological shortcomings.^29^

### Inclusion Criteria

Included studies met the following criteria: (1) all participants had a confirmed diagnosis of a psychotic disorder according to recognized diagnostic criteria (eg, *Diagnostic and Statistical Manual of Mental Disorder*); (2) reported cross-sectional zero-order bivariate correlations between all 3 outcomes of interest (ie, EP, symptoms, and social functioning); (3) written in English; and (4) published in peer-reviewed academic journals.

### Stage of Illness

The cohorts included in the review were classified according to stage of illness: FEP or established psychosis. FEP cohorts comprised of individuals experiencing a confirmed first episode of a psychotic disorder or with a duration of illness of less than 5 years. This cut-off was chosen because illness presentation can fluctuate considerably during the first 5 years, after which illness course tends to be more stable.^30^ Cohorts with a duration of psychotic illness greater than 5 years, or who did not clearly meet our criteria for FEP, were assigned to the established psychosis group; this included some studies that had a mix of individuals experiencing FEP or longer-standing psychosis.

### Outcomes

The definition of EP provided by the social cognition psychometric evaluation (SCOPE) study^31^ was used to guide the selection of EP tasks and to differentiate between lower- and higher-level tasks. Lower-level EP tasks were those involving identifying or recognizing emotions from facial, voice, or body cues (eg, Bell and Lysaker Emotion Recognition Task [BLERT]^32^) and higher-level EP tasks focused on testing an individual’s ability to understand or manage emotions (eg, Mayer-Salovey-Caruso Emotional Intelligence Test—Managing Emotions branch [MCSCEIT-ME]^33^).

The analysis focused on 4 symptom domains: positive, negative, disorganization, and depressive symptoms. “*Positive symptoms*” refers to hallucinations and delusions, as measured by factor scores, summed items, or subscale scores on structured psychotic symptom measures (eg, Positive and Negative Syndrome Scale [PANSS]^34^). Measurement of positive symptoms is complicated by the inclusion of disorganization items in some scales (eg, “conceptual disorganization” in the PANSS positive subscale). In an inclusive approach, scores derived from total positive subscale scores were classified as “*positive symptoms.” To explore disorganization as a potential confound, the approach of Ventura et al.*^19^ was followed, dividing positive symptom scales into those that solely measured reality distortion symptoms (ie, hallucinations and delusions) and those that combined reality distortion and disorganization symptoms, for the purpose of sensitivity analysis. “*Negative symptoms*” refers to items assessing anhedonia, avolition, asociality, alogia, and blunted affect. “*Disorganization*” refers to ratings of disorganized thinking and behavior and/or cognitive symptoms. For “*depressive symptoms*,” validated depression measures (eg, Calgary Depression Scale for Schizophrenia [CDSS]^35^), as well as factor-derived scores from psychotic symptom scales (eg, the “depression/anxiety” factor derived from Wallwork’s^36^ 5-factor solution of PANSS), were included, though again these were considered separately in the sensitivity analysis.

There is notable variability in the definition and measurement of social functioning.^37^ For this study we were specifically interested in direct real-world functional outcomes rather than task-based performance in social situations (ie, functional capacity). To aid the selection of social functioning measures, we used the approach of several previous reviews^9,11,13^ to classify measures into four domains of functioning: community functioning (ie, functional outcomes in everyday life), social behavior in the milieu (ie, observed behavior in treatment settings^9^), social skills, and social problem solving (the latter two which are considered aspects of functional capacity^13^). We included only community functioning measures (eg, Personal and Social Performance Scale [PSP]^38^).

### Study Selection

The screening process was undertaken using Covidence.^39^ In the first phase, titles, and abstracts were screened to exclude studies that clearly: (1) were the incorrect article type (eg, book chapter); (2) did not include individuals with a psychotic disorder; or (3) did not include a measure of social cognition and either symptoms or social functioning. In the second phase, full-text articles were screened against the inclusion criteria stated above. In both phases, screening was conducted independently by the lead author and one other reviewer, with disagreements resolved via consensus-based discussion. Decision flowcharts were used to guide decision-making processes. Agreement was >80% at title and abstract screening and >97% at full-text screening.

The third phase involved the management of overlapping cohorts. Probable overlapping cohorts were identified by cross-referencing authors, research groups, grant numbers, or ethics approvals. The authors were contacted to seek clarification where this was unclear. The inclusion of such publications was decided on a case-by-case basis, accounting for the number of unique correlations reported and cohort size.

The final phase involved contacting authors of studies published in the past 5 years that met all criteria except reporting correlations between all 3 outcomes of interest. Authors were requested to provide the appropriate correlations. The authors were not contacted if the study cohort overlapped with an already included cohort. A cut-off date of 5 years was used due to the reduced likelihood of success in contacting authors of older papers and their likely ability to access data.

### Data Extraction

Data were extracted from included studies independently by the lead author and one other reviewer using a purpose-designed extraction form. Extracted information included characteristics of the study (eg, country, setting) and cohort (eg, age, diagnoses), details of outcome measures, and correlations between outcomes of interest (see Table S5 for a comprehensive list). Data were extracted for all relevant outcome measures where multiple were available (eg, 2 or more EP tasks were used). Where studies provided data for multiple separate cohorts at different stages of illness (ie, FEP and established psychosis), data were extracted separately for each cohort. Any disagreements were resolved via consensus discussion between authors.

### Appraisal of Methodological Quality

Methodological quality was assessed for each included study independently by the lead author and one other reviewer using a checklist adapted from the Joanna Briggs Institute (JBI) Critical Appraisal Checklist for Analytical Cross-Sectional Studies^40^ (Table S6). The adapted checklist contained 7 items related to methodological quality of study inclusion criteria, description of study subjects/setting, measurement of psychosis, measurement of outcomes (3 items), and statistical analysis. A response of “yes” (scored 1), “no,” or “unclear” (both scored 0) was given for each item and scores were tallied to provide a score out of 7 for each study, with higher scores indicating greater methodological rigor. Any disagreements were resolved via consensus discussion between authors. Agreement for methodological quality item ratings across included studies was 87%. All studies were included regardless of methodological quality; however, this rating scale was used in moderator analyses.

### Statistical Analysis

For each study, the extracted effects included at least 3 cross-sectional correlations between (1) EP and available symptom domains; (2) EP and social functioning; and (3) social functioning and available symptom domains, resulting in nine correlation pairs. Where studies reported more than one correlation for each coded relationship, a mean correlation was calculated so that studies were represented only once for each relationship, following the approach used in previous reviews.^19,41^

Statistical analysis was conducted in R (version 4.3.1),^42^ following recommendations from Harrer et al.^43^ First, we examined correlations between each of the outcome pairs in the total sample. Using the “*metafor*” package (version 4.2.0),^44^ random effects meta-analyses were conducted to calculate effect sizes and 95% confidence intervals for each of the correlation pairs where *k* ≥ 3. Correlations were converted to Fisher’s *z* to pool effect sizes and calculate confidence intervals before being reconverted to *r*. Cohen’s conventions were used to interpret the strength of the correlations, where *r* ≈ .1 was considered a small effect, *r* ≈ .3 moderate, and *r* ≈ .5 large.^45^ Between-study heterogeneity was examined using *I*^2^ and *Q* statistics. Higgins and Thompson’s^46^ guidelines for interpretation of *I*^2^ statistic were used, where *I*^2^ = .25 indicates low heterogeneity, *I*^2^ = .50 moderate heterogeneity, and *I*^2^ = .75 substantial heterogeneity. Publication bias was assessed by examining funnel plots and calculating Egger’s regression test.

Next, we examined the moderators of these relationships. The total sample was separated into groups classified according to the stage of illness (ie, FEP, established psychosis) and subgroup analyses were conducted to compare effect sizes across illness stages. Cohorts were classified according to EP task type and subgroup analyses were conducted for lower- versus higher-level EP tasks; as mean correlations were used for each cohort, those that included both task types were excluded from this analysis. Exploratory mixed-effect model meta-regression analyses were also conducted to examine the moderating effects of demographic and clinical variables including age, gender, diagnosis, and setting. Although corrections for multiple comparisons reduce the risk of Type I errors, they can inflate the risk of Type II errors (1−β).^47^ Due to the exploratory nature of these analyses and to minimize loss of statistical power, alpha (α) was unadjusted and set at the .05 level for subgroup and moderator analyses.

Mediation analyses were conducted to test whether each symptom domain-mediated the relationship between EP and social functioning, using the “*metaSEM*” package (version 1.3.1)^48^ and incorporating a 2-stage meta-analytic structural equation modeling approach (MASEM). First, correlations were pooled across studies using random effect models; second, weighted least square (WLS) estimation was used to fit the proposed mediation model to the pooled correlation matrix. Mediation analysis provided estimates and 95% confidence intervals of the partially direct effect of EP on social functioning, and an indirect effect of EP on social functioning through the symptom domain. We were primarily interested in the indirect effect: a non-zero indirect effect with a 95% confidence interval not capturing zero was considered as evidence of a significant mediating effect.

Sensitivity analyses were conducted on meta-analyses and, where appropriate, mediation analyses, to assess the robustness of the findings to decisions related to classification of outcome measures and potential outliers. Where possible, exploratory analyses were conducted to assess relationships between symptoms and both EP and social functioning with the more fine-grained assessment of symptoms (eg, subdomains of negative symptoms).

## Results

### Included Studies

**Figure 1.**
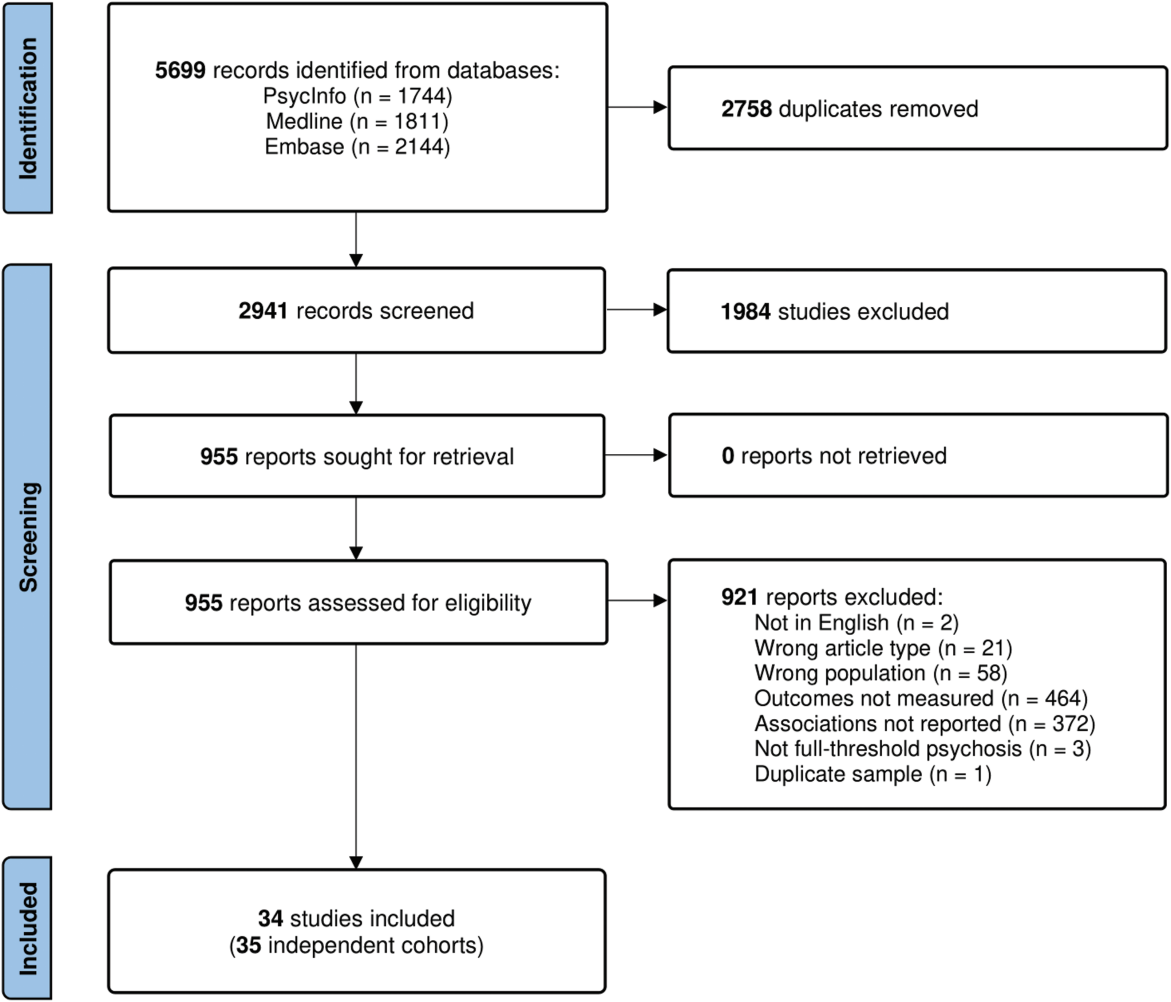
PRISMA Flow Diagram

The literature search and screening process resulted in the inclusion of 34 independent studies (see Figure 1 for PRISMA flowchart). One study^49^ included two distinct cohorts at different stages of illness, leading to 35 independent cohorts (referred to as *k*) in the analysis: four FEP, and 31 established psychosis. One study was excluded due to an overlapping cohort,^50^ and a further two^51,52^ that otherwise appeared to meet criteria were excluded due to insufficient information regarding the reported correlations (see Table S7).

Characteristics of the included cohorts and the outcome measures used are presented in Table 1, and a summary of the included measures for each outcome can be found in Tables S8-S10. The total sample included 3304 participants (65% male), with an average age of 37.48 years. The 4 FEP cohorts comprised 327 participants with an average age of 23.94 years and 61% were male. Duration of illness was only reported for 2 FEP studies, with an average duration of illness of 1.65 years. Within the FEP group, 42% had a diagnosis of schizophrenia, 14% were diagnosed with schizophreniform or brief psychotic disorder, and the remainder (44%) had a diagnosis of schizoaffective or another psychotic disorder. There were 2977 participants (65% male) in the established psychosis group, most (92%) with a diagnosis of schizophrenia, with an average age of 38.96 and an average illness duration of 14.88 years.

**Table 1.** Characteristics of Included Studies and the Outcomes Measures Used

Study	Setting	*n*	Age	Male (%)	Illness duration (yrs)	Diagnoses	AP dose (CPZ)	EP measures	Symptom measures	Social functioning measures	Quality (/7)
First Episode Psychosis											
Brasso et al., 2023^a^^49^	OP	75	27.3	78.7	2.3	SZ: 100%	—	MSCEIT-ME	pos—PANSS-5 factor^b^ neg—BNSS dis—PANSS-5 factor^b^ dep—CDSS	SLOF	7
Gonzalez-Blanch et al., 2020^53^	OP	170	20.90	52.90	—	SZ: 17.1%, Schizophreniform/BPD: 12.4%; SA: 11.2%, NOS: 17.6%, Affective psychoses: 34.1%, DD: 2.9%, SIP: 4.7%	—	BLERT	pos—PANSS subscale neg—PANSS subscale	PSP	6
Haining et al., 2022^a^^54^	OP	15	24.40	53.33	—	SZ: 66.7%, Schizophreniform: 6.7%, SA: 6.7%, NOS: 20%	—	ER-40	pos—CAARMS subscale	GAF	7
Larsson et al., 2022^a^^55^	—	67	27.80	61.19	0.93	SZ: 32.8%, Schizophreniform/BPD: 35.8%, SA: 3.0%, NOS: 23.9%, DD: 4.5%	—	FEFA-2	pos—PANSS subscale neg—PANSS subscale	GAF	7
Established psychosis											
Abram et al., 2014^56^	OP	59	35.51	62.70	14.77	SZ: 100%	363.30	Facial Affect Perception task	pos—SAPS (hallucinations/delusions items) neg—SANS (affective flattening, alogia, avolition, anhedonia items) dis—SAPS/SANS (positive formal thought disorder, bizarre behavior, attention items)	SLOF	7
Brasso et al., 2023^a^^49^	OP	92	43.5	64.1	20	SZ: 100%	—	MSCEIT-ME	pos—PANSS-5 factor^b^ neg—BNSS dis—PANSS-5 factor^b^ dep—CDSS	SLOF	7
Brittain et al., 2012^57^	OP	64	41.89	53.13	18.41	SZ: 100%	461.95	FAR task/EmoBio	pos—PANSS subscale neg—PANSS subscale	RFS	6
Brown et al., 2014^58^	OP	45	36.16	51.11	12.17	SZ: 100%	488.91	FEIT/FEDT	pos—PANSS subscale neg—PANSS subscale	SFS	7
Charernboon et al., 2021^a^^59^	OP	64	37.00	42.20	8.00	SZ: 100%	—	Faces test	pos—SAPS neg—SANS (—attention items)	PSP	6
Cohen et al., 2006^60^	IP	28	33.36	85.71	—	SZ: 100%	—	FEIT	pos—BPRS positive factor^c^ neg—BPRS negative factor^c^ dis—BPRS disorganization factor^c^	SAS-II	7
Davidson et al., 2018^61^	OP	48	51.00	58.30	—	SZ: 77.2%, SA: 12.5%, other: 10.3 %	—	BLERT/MSCEIT-ME	pos—PANSS-5 factor^d^ neg—PANSS-5 factor^d^ dis—PANSS-5 factor^d^ dep—PANSS-5 factor^d^	QLS	6
Engelstad et al., 2017^62^	IP/OP	54	28.70	61.11	5.80	SZ: 70.4%, SA: 29.6%	—	EmoBio	pos—PANSS-5 factor^b^ neg—PANSS-5 factor^b^ dis—PANSS-5 factor^b^ dep—PANSS-5 factor^b^	SFS	6
Erol et al., 2009^63^	OP	100	35.90	58.00	13.10	SZ: 100%	—	FEIT/FEDT	pos—SAPS neg—SANS dep—CDSS	SFS	6
Farina et al., 2022^a^^64^	OP	52	25.66	67.30	—	SZ: 100%	367.19	BLERT	dep—BDI-II	SFS/QLS	6
Fiszdon et al., 2023^a^^65^	OP	51	47.8	61	—	SZ: 84.4% SA: 15.6%	—	FEIT/MSCEIT-ME	pos—PANSS-5 factor^d^ neg—PANSS-5 factor^d^ dis—PANSS-5 factor^d^ dep—PANSS-5 factor^d^	ILSS/QLS—Interpersonal Relations/SFS	7
Galderisi et al., 2018^66^	OP	740	40.00	70.10	16.40	SZ: 100%	—	FEIT/MSCEIT-ME	pos—PANSS-5 factor neg—BNSS dis—PANSS (conceptual disorganization item) dep—CDSS	SLOF	7
Grant et al., 2010^67^	OP	123	38.60	65.80	—	SZ: 82.1%, SA: 17.9%	—	ER-40/Penn Emotion Discrimination Task	pos—SAPS neg—SANS (—attention, inappropriate affect items) dep—BDI-II	SFS	4
Hajduk et al., 2020^a^^68^	—	59	39.29	56.00	—	SZ or SA: 100%	—	ER-40/BR-100	pos—PANSS subscale neg—PANSS subscale	PSP/SLOF—Interpersonal Relationships and Social Appropriateness	5
Hajduk et al., 2018^69^	OP	43	38.16	60.47	11.30	SZ: 72.1%, SA: 27.9%	—	Emotion Recognition Task	pos—CGI-SCH subscale neg—CGI-SCH subscale dis—CGI-SCH subscale dep—CGI-SCH subscale	PSP	5
Huang et al., 2017^70^	IP/OP	74	33.22	41.89	10.65	SZ: 100%	445.12	CFERD	pos—PANSS subscale neg—PANSS subscale dep—CDSS	SFS	7
Kalin et al., 2015^71^	OP	179	42.11	65.36	—	SZ: 54%, SA: 46%	—	BLERT/ER-40	neg—PANSS-5 factor^e^	SLOF -Interpersonal Functioning	7
Kolavarambath et al., 2020^72^	IP/OP	27	31.07	51.85	7.00	SZ: 100%	—	TRENDS/TAS	pos—SAPS neg—SANS	GSDS	6
Lecomte et al., 2019^a^^73^	OP	47	38.00	62.00	—	SZ: 100%	—	FEIT/FEDT	pos—BPRS subscale neg—BPRS subscale	SFS	5
Lin et al., 2013^17^	IP/OP	302	38.17	61.26	14.47	SZ: 100%	495.67	MSCEIT-ME	pos—PANSS subscale neg—SANS dep—HAMD	GAF/QLS	7
Nijman et al., 2023^a^^74^	IP/OP	81	37.78	69.14	12.63	SZ: 60.5%, SA: 22.2%, other: 17.3%	—	FEEST	pos—PANSS subscale neg—PANSS subscale dep—BDI-II	PSP	6
O’Reilly et al., 2015^75^	IP/OP	89	40.36	94.38	—	SZ: 85.4%, SA: 14.6%	564.29	MSCEIT-ME	pos—PANSS subscale neg—PANSS subscale	SOFAS	6
Ospina et al., 2019^a^^76^	OP	45	44.51	53.30	—	SZ: 51.1%, SA: 48.9%	—	ERT (CANTAB)/MSCEIT-ME/TAS	dep—HAMD	WHODAS 2.0/SAS-SR	7
Pijnenborg et al., 2009^77^	IP/OP	46	27.40	73.91	7.00	SZ: 100%	—	Prosody task/FEEST	pos—PANSS-5 factor^f^ neg—PANSS-5 factor^f^ dis—PANSS-5 factor^f^ dep—PANSS-5 factor^f^	SFS	5
Riccardi et al., 2016^78^	OP	30	37.80	70.00	13.87	SZ: 100%	—	Pictures of Facial Affect Task	pos—PANSS-5 factor^d^ neg—PANSS-5 factor^d^ dis—PANSS-5 factor^d^	GAF	4
Sampedro et al., 2021^79^	IP/OP	96	41.37	88.54	18.59	SZ: 100%	511.37	BLERT	pos—PANSS-5 factor^b^ neg—PANSS-5 factor^b^ dis—PANSS-5 factor^b^ dep—PANSS-5 factor^b^	SFS	5
Shen et al., 2022^a^^80^	IP	76	30.84	65.79	—	SZ: 100%	—	C-FAIT	pos—PANSS subscale neg—PANSS subscale	PSP	6
Tabak et al., 2015^81^	OP	35	47.06	60.00	—	SZ: 100%	—	TMMS	pos—BPRS subscale neg—CAINS dep—HAMD	RFS	7
Tost et al., 2023^a^^82^	—	38	35.00	76.32	8.40	SZ: 100%	460.00	MSCEIT-ME	pos—PANSS subscale neg—PANSS subscale dep—CDSS	PSP	7
Tso et al., 2010^83^	OP	33	38.50	66.67	17.90	SZ or SA: 100%	505.10	MSCEIT-ME	pos—BPRS (hallucinatory behavior, unusual thought content, suspiciousness, conceptual disorganization items) neg—BPRS (emotional withdrawal, motor retardation, flat affect items)	GAF/SAS-SR	7
Zhu et al., 2020^a^^84^	OP	157	43.69	54.14	18.61	SZ: 100%	266.01	MSCEIT	pos—PANSS subscale neg—PANSS subscale	PSP	6

Abbreviations: BDI, Beck Depression Inventory; BLERT, Bell Lysaker Emotion Recognition Task; BNSS, Brief Negative Symptoms Scale; BPD, brief psychotic disorder; BPRS, Brief Psychiatric Rating Scale; BR-100, Body Emotion Recognition Task; CAARMS, Comprehensive Assessment of At-Risk Mental State; CAINS, Clinical Assessment Interview for Negative Symptoms; CDSS, Calgary Depression Scale for Schizophrenia; C-FAIT, Chinese version of the Face-Affective Identification Task; CFERD, Chinese Facial Emotion Recognition Database; CGI-SCH, Clinical Global Impression—Schizophrenia Scale; CPZ, chlorpromazine equivalents; DD, delusional disorder; dep, depressive symptoms; dis, disorganization symptoms; EmoBio, Emotion in Biological Motion; EP, emotion processing; ER-40, Penn Emotion Recognition Task; ERT (CANTAB), Emotion Recognition Task from Cambridge Neuropsychological Test Automated Battery; FAR, facial affect recognition; FEEST, Facial Expression of Emotions: Stimuli and Test; FEFA-2, Frankfurt Test and Training of Facial Affect Recognition 2nd Edition; FEDT, Face Emotion Discrimination Task; FEIT, Face Emotion Identification Task; GAF, Global Assessment of Functioning; GSDS, Groningen Social Disabilities Schedule; HAMD, Hamilton Depression Rating Scale; ILSS, Independent Living Scales Survey; IP, inpatient; MSCEIT-ME, Mayer-Salovey-Caruso Emotional Intelligence Test—Managing Emotions; neg, negative symptoms; NOS, psychotic disorder not otherwise specified; OP, outpatient; PANSS, Positive and Negative Syndrome Scale; pos, positive symptoms; PSP, Personal and Social Performance Scale; QLS, Quality of Life Scale; RSF, Role Functioning Scale; SA, schizoaffective disorder; SANS, Scale for the Assessment of Negative Symptoms; SAPS, Scale for the Assessment of Positive Symptoms; SAS, Social Adjustment Scale; SAS-SR, Social Adjustment Scale-Self Report; SFS, Social Functioning Scale; SIP, substance-induced psychotic disorder; SLOF, Specific Level of Functioning Scale; SOFAS, Social and Occupational Functioning Assessment Scale; SZ, schizophrenia; TAS, Toronto Alexithymia Scale; TMMS, Trait-Meta Mood Scale; TRENDS, Tool for Recognition of Emotions in Neuropsychiatric Disorders; WHODAS, World Health Organization Disability Assessment Schedule.

^a^Correlations provided by the author on request.

^b^5-factor solution from Wallwork et al.^36^

^c^4-factor solution from Meuser et al.^85^

^d^5-factor solution from Bell et al.^86^

^e^5-factor solution from Marder et al.^87^

^f^5-factor solution from Lindenmayer et al.^88^

### Associations Between Correlation Pairs

**Figure 2.**
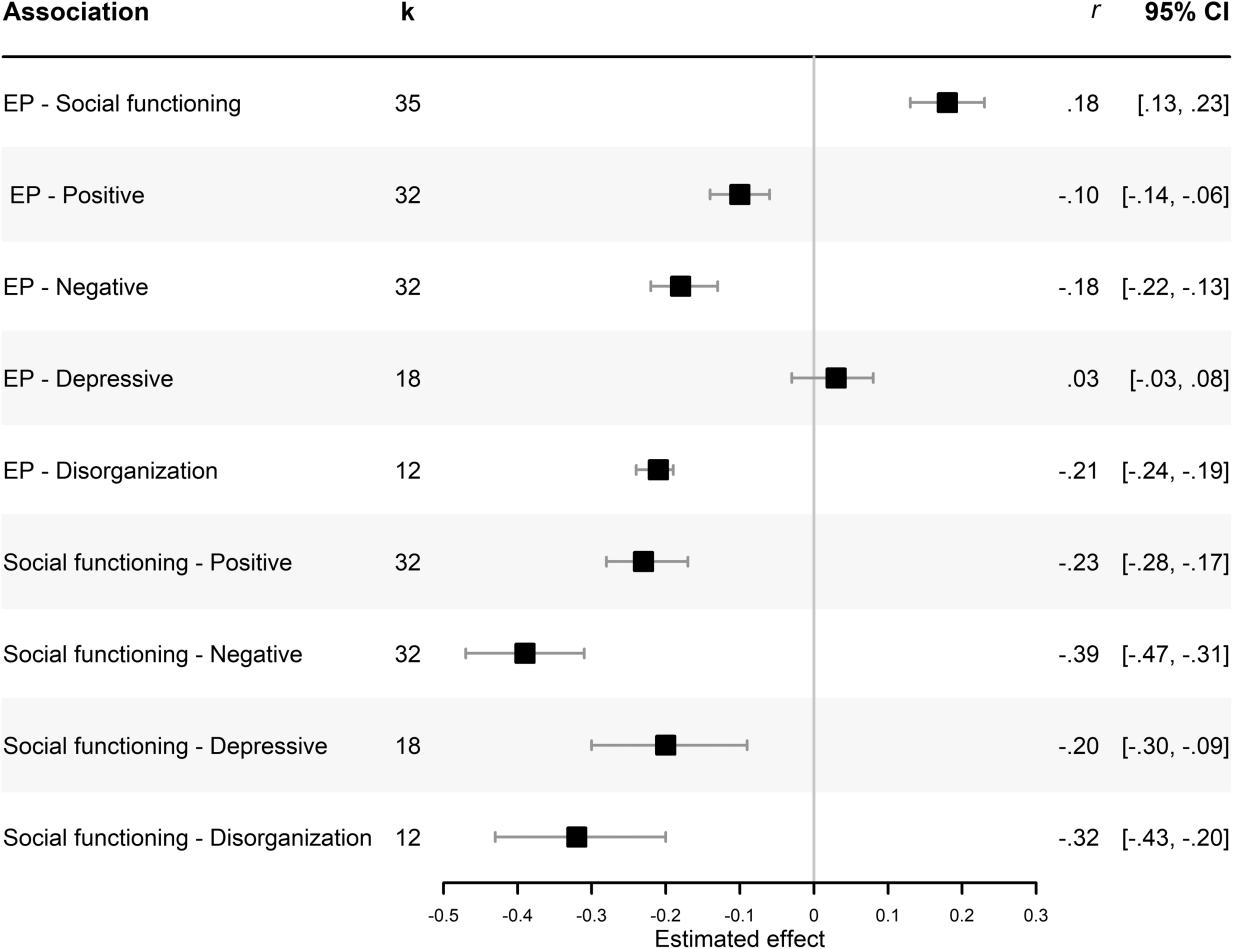
Forest Plots Displaying Estimated Effect Sizes and 95% Confidence Intervals Obtained From Meta-analyses of Associations Between Outcome Variables

Estimated effect sizes obtained from the meta-analyses for the 9 correlation pairs are presented in Figure 2. The forest plots for each of the meta-analyses can be found in Supplementary Materials.

There was a small to moderate positive relationship between EP and social functioning (*r* = .18, 95% CI: [0.13, 0.23]), with low to moderate heterogeneity between studies (*I*^2^ = 36.67%, *Q* = 53.68, *P* = .017). For symptoms, the strongest relationship was found between EP and the disorganization symptom domain (*r* = −.21, 95% CI: [−0.24, −0.19]), followed by negative symptoms (*r* = −.18, 95% CI: [−0.22, −0.13]) and positive symptoms (*r* = −.10, 95% CI: [−0.14, −0.06]). The estimated association between EP and depressive symptoms was very small and not statistically significant (*r* = .03, 95% CI: [−0.03, 0.08]). Between-study heterogeneity for all EP-symptom correlations was low (all *I*^2^ < 25%).

Social functioning was most strongly associated with the negative symptom domain (*r* = −.39, 95% CI: [−0.47, −0.31]), followed by disorganization symptoms (*r* = −.32, 95% CI: [−0.43, −0.20]), positive symptoms (*r* = −.23, 95% CI: [−0.28, −0.17]), and depressive symptoms (*r* = −.20, 95% CI: [−0.30, −0.09]). There was moderate or substantial between-study heterogeneity in most symptom-social functioning correlations: social functioning and negative symptoms (*I*^2^ = 80.08%, *Q* = 155.62, *P* < .001); social functioning and depressive symptoms (*I*^2^ = 72.77%, *Q* = 62.44, *P* < .001); and social functioning and disorganization symptoms (*I*^2^ = 61.15%, *Q* = 28.31, *P* = .003). Between-study heterogeneity in the social functioning-positive symptoms correlation was low to moderate (*I*^2^ = 38.68%, *Q* = 50.56, *P* = .015).

### Moderator Analyses

Results of the subgroup analyses for stage of illness and EP task type are presented in Table 2.

**Table 2.** Subgroup Analyses for Stage of Illness and Task Type

Association/moderator	*k*	*r*	95% CI	*P*	*I^2^*	*P*_subgroup_
EP—Social functioning						
Stage of illness						
FEP	4	.09	[−.01, .20]	.066	0.00%	.026
Established	31	.19	[.13, .24]	<.001	40.10%	
Task Type						
Lower-level EP	22	.16	[.08, .23]	<.001	47.70%	.091
Higher-level EP	8	.24	[.16, .32]	<.001	1.40%	
EP—Positive symptoms						
Stage of illness						
FEP	4	.01	[−.08, .10]	.703	0.00%	.001
Established	28	−.12	[−.16, −.07]	<.001	7.30%	
Task Type						
Lower-level EP	20	−.11	[−.17, −.06]	<.001	0.00%	.683
Higher-level EP	8	−.09	[−.22, .06]	.197	50.10%	
EP—Negative symptoms						
Stage of illness						
FEP	3	−.19	[−.57, .26]	.219	61.20%	.921
Established	29	−.18	[−.22, −.13]	<.001	21.80%	
Task Type						
Lower-level EP	20	−.15	[−.21, −.08]	<.001	25.80%	.004
Higher-level EP	8	−.26	[−.32, −.20]	<.001	0.00%	
EP—Depressive symptoms						
Stage of illness	Not conducted, as *k* < 3 for FEP					
Task Type						
Lower-level EP	9	.00	[−.09, .08]	.900	0.00%	.239
Higher-level EP	5	.08	[−.13, .28]	.360	63.30%	
EP—Disorganization symptoms						
Stage of illness	Not conducted, as *k* < 3 for FEP					
Task Type	Not conducted, as *k* < 3 for higher-level EP					
Social functioning—Positive symptoms						
Stage of illness						
FEP	4	−.33	[−.50, −.13]	.014	27.60%	.071
Established	28	−.21	[−.27, −.15]	<.001	37.40%	
Social functioning—Negative symptoms						
Stage of illness						
FEP	3	−.47	[−.63, −.28]	.011	0.00%	.136
Established	29	−.38	[−.57, −.29]	<.001	81.60%	
Social functioning—Depressive symptoms						
Stage of illness	Not conducted, as *k* < 3 for FEP					
Social functioning—Disorganization symptoms						
Stage of illness	Not conducted, as *k* < 3 for FEP					

Abbreviations: EP, emotion processing; FEP, first episode psychosis.

#### Stage of Illness Subgroup Analysis.

The estimated association between EP and social functioning was smaller in FEP (*r* = .09) than in established illness (*r* = .19), *P* = .026. The estimated strength of association between EP and positive symptoms was also smaller in FEP (*r* = .01) than in established illness (*r* = −.12), *P* = .001. No significant differences were found for the relationships between EP and negative symptoms, social functioning and positive symptoms, or social functioning and negative symptoms (all *P* > .05). As disorganization and depressive symptoms were only measured in one FEP cohort, associations involving these symptoms were not examined for this analysis.

#### EP Task Level Subgroup Analysis.

The negative association between EP and negative symptoms was stronger for higher-level (*r* = −.26) compared with lower-level EP tasks (*r* = −.15); this difference was statistically significant (*P* = .004). No other differences between EP task levels were found (all *P* > .05).

#### Exploratory Moderator Analyses.

Given the high between-study heterogeneity in several meta-analyses, exploratory moderator analyses were conducted on the 9 correlation-pair meta-analyses (Table S11). The variables included were age, gender, study setting (ie, inpatient vs outpatient), diagnosis (ie, schizophrenia vs psychosis spectrum), and methodological quality (as assessed in the quality appraisal). Diagnosis was the only significant moderator: in exclusively schizophrenia cohorts compared with those that included a broader range of psychotic disorders, there was a weaker negative relationship between EP and disorganization and a weaker negative relationship between social functioning and depressive symptoms. The moderating effects of diagnosis explained less than 1% of between-study heterogeneity for the correlation between EP and disorganization symptoms and 33.81% of between-study heterogeneity for the social functioning and depressive symptoms correlation.

### Mediation Analyses

**Figure 3.**
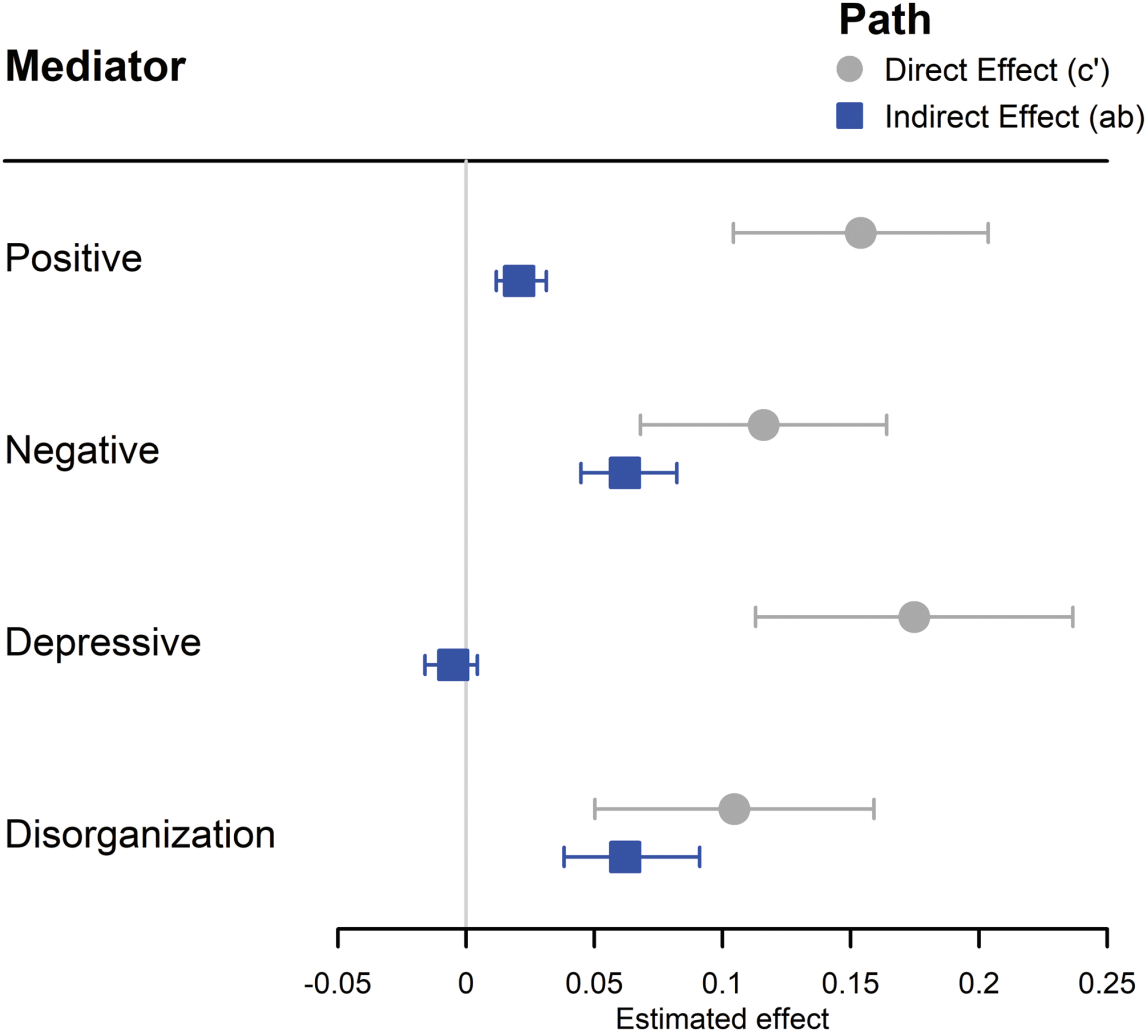
Forest Plots Displaying the Results of the Mediation Analyses Examining the Symptom Domains as Mediators of the Relationship Between EP and Social Functioning. *Note*: The Direct Effect (*cʹ*) Represents the Direct Path From EP to Social Functioning, Adjusted for the Mediating Effect. The Indirect Effect (ab) Represents the Mediating Effect From EP to Social Functioning Through the Symptom Domain. A Non-zero Indirect Effect Indicates a Significant Mediating Effect

The results of the mediation analyses are presented in Figure 3. The relationship between EP and social functioning was partially mediated by all symptom domains except for depressive symptoms, though estimated effect sizes were small. The indirect effect was larger through negative and disorganization symptoms (β_indirect_ = .06, 95% CI: [0.04, 0.08] and β_indirect_ = .06, 95% CI: [0.04, 0.09], respectively) than positive symptoms (β_indirect_ = .02, 95% CI: [0.01, 0.02]).

### Exploratory Analysis

While studies mostly presented correlations with total symptom domain scores, rather than more specific symptoms (eg, hallucinations), there was data available from a limited number of cohorts (*k* = 4) that divided negative symptoms into 2 factors: an experiential factor (encompassing avolition, asociality, and anhedonia), and an expressive factor (encompassing blunted affect, and alogia). In exploratory meta-analyses (Table S12), EP was negatively associated with expressive negative symptoms (*r* = −.21, 95% CI: [−0.34, −0.08]), but the association between EP and experiential symptoms was not statistically significant (*r* = −.16, 95% CI: [−0.36, 0.06]). Social functioning was negatively associated to a similar degree with both expressive (*r* = −.39, 95% CI: [−0.56, −0.18]) and experiential negative symptoms (*r* = −.40, 95% CI: [−0.53, −0.25]).

### Sensitivity Analyses

Sensitivity analyses were conducted on the relevant meta-analyses to assess the robustness of the findings to decisions regarding the classification of symptoms, social functioning, and stage of illness, and to the effects of potential outliers (see Supplementary Materials for full account). Results were broadly robust with several notable findings. The inclusive approach we took to the classification of the outcomes in the primary analyses had limited impacts on estimated effects with one exception: the negative relationship between positive symptoms and social functioning was estimated to be weaker for reality distortion-only measures (*r* = −.17), compared with disorganization only measures (*r* = −.26). Notably, when only those EP measures considered to have good psychometric properties as per the SCOPE validation study^89^ (ie, BLERT and Penn Emotion Recognition Task) plus the MSCEIT-ME were included, similar effect sizes were estimated for the correlations between EP and both social functioning and the symptom domains. Finally, the removal of potential outliers did not substantially change the findings.

Additional sensitivity analyses using mediation models were conducted (see Supplementary Materials). When measures that encompassed disorganization items were excluded, the mediating effect of positive symptoms (ie, reality distortion) on the relationship between EP and social functioning was smaller but remained significant (β_indirect_ = .01, 95% CI: [0.005, 0.03]). We also tested mediation by negative symptoms for lower- and higher-level tasks separately. Negative symptoms mediated the relationship between lower-level EP tasks and social functioning (β_indirect_ = .05, 95% CI: [0.03, 0.07]) and between higher-level EP tasks and social functioning (β_indirect_ = .09, 95% CI: [0.06, 0.13]).

### Publication Bias

Publication bias was assessed by Egger’s regression test and examination of funnel plots for each of the meta-analyses (see Supplementary Materials). There was no evidence of publication bias; however, Egger’s test may lack the power to detect bias when study numbers are small.^43^ Evidence from Egger’s regression tests was therefore corroborated by examination of funnel plots, with no clear funnel plot asymmetry found. In sum, there is no clear evidence to suggest that estimated effects are impacted by publication bias.

## Discussion

The principal aims of this systematic review and meta-analysis were to examine the interrelationships between EP, social functioning, and symptoms in psychotic disorders, to test whether symptom domains mediated the relationship between EP and social functioning, and to explore the moderating effects of stage of illness and EP task type (ie, lower- and higher-level EP) on these relationships. There was a significant but small relationship between EP and social functioning which was mediated by negative and disorganization symptoms and, to a lesser degree, positive symptoms. Contrastingly, depressive symptoms were not significantly associated with EP and did not mediate the relationship between EP and social functioning. The strength of the relationship between EP and both social functioning and positive symptoms was weaker in FEP cohorts compared with established illness cohorts. Finally, there was a stronger relationship between negative symptoms and higher-level than lower-level EP tasks.

### Role of Symptoms in the EP Social Functioning Relationship

Previous meta-analyses have demonstrated that EP is independently associated with both symptoms (including positive, negative, and disorganization symptoms) and functioning.^13,19^ To our knowledge this is the first meta-analysis showing that positive, negative, and disorganization symptoms partially mediate the relationship between EP and social functioning.

The findings provide support for the hypothesis that impairments in social cognitive processes play a role in the development of symptoms,^15,90^ which could then have downstream effects on social functioning. For example, the mediating role of disorganization symptoms suggests that difficulties accurately perceiving others’ emotions could play a role in the failure to effectively adapt during social interaction, manifesting as speech or behavior that is interpreted as disorganized. Similarly, difficulties in recognizing another’s facial expressions could impair a person’s ability to mirror that facial expression, resulting in blunted affect, which in turn limits efficacy in social interactions. It is noted that the indirect effects were smaller than the direct effects of EP on social functioning. This suggests that EP may also influence social functioning directly, or there are mechanisms not considered here, such as sensorimotor processing deficits, that could underly both EP and symptom deficits and influence social functioning.^15^

Disorganization and negative symptoms had a larger estimated indirect effect of EP on social functioning than positive symptoms, suggesting that these domains are most important for understanding the links between EP and social functioning in psychotic illness. Overall, the pattern of findings is consistent with previous reviews examining relationships between symptom domains and social functioning,^91^ and EP and symptom domains.^19^ As noted previously,^91^ these effects may be party related to some conceptual overlap between items of negative symptom measures (eg, “emotional withdrawal” and “passive/apathetic social withdrawal items” of the PANSS) with social functioning measures. Similarly, symptom scale items related to “attention” or “abstract thinking,” which may be classified as negative or disorganization symptoms, may tap into cognitive abilities that are required for optimal performance on EP tasks.

A stronger relationship was found between EP and disorganization compared with EP and positive symptoms, and when positive symptom measures that encompassed items assessing disorganization were removed in sensitivity analyses, the relationship between EP and positive symptoms was further reduced. This is consistent with outcomes of a previous systematic review,^92^ which also highlighted disorganization as a distinct symptom domain, and an important independent factor in the relationship between EP and social functioning. Notably, however, disorganization symptoms were much less likely to be assessed than positive or negative symptoms.

The current findings corroborate those of previous meta-analyses, which have demonstrated small to moderate associations between EP and negative symptoms.^4,19^ Our exploratory analysis provides novel findings that EP is associated with the expressive negative symptom subdomain (encompassing blunted affect and alogia) but not the experiential negative symptom subdomain (encompassing avolition, asociality, and anhedonia). Although results should be interpreted with caution given the small subset of studies included this analysis, they are important: if the negative symptom subdomains are differentially related to social cognition, using summed negative symptom domains may reduce observed correlations with other variables such as EP. Furthering our understanding of the links between social cognition and the subdomains may provide greater insights into the mechanisms underlying their relationships.

Depressive symptoms were related to social functioning but were not related to EP. This highlights the importance of recognizing and treating depressive symptoms in order to improve social functioning in psychosis,^93^ but also suggests that the link between EP and social functioning occurs through mechanisms independent of the relationship between depression and social functioning. As well as the direct effects of depressive symptoms such as fatigue or hopelessness on social functioning, depression may be linked to other personal, and interpersonal factors such as loneliness, social withdrawal, or self-esteem,^94^ which are often underconsidered.^95^

### Moderating Effects of Illness Stage

A surprisingly small number of FEP (*n* = 4) studies met inclusion criteria for the review and a smaller number of these included measures of depression (*n* = 1) or disorganization (*n* = 1), limiting comparisons across stages of illness to the positive and negative symptom domains. Despite this, there were some observed differences across illness stages: the strength of associations between EP and social functioning and EP and positive symptoms were smaller in studies with FEP cohorts than those with established illness cohorts.

It is possible that reduced variability and lower severity across EP impairment, positive symptoms, and social dysfunction in FEP, could truncate observed correlations. There is some evidence that social cognition is worse in individuals with unremitted, compared with remitted, schizophrenia,^96^ thus severe or unremitted positive symptoms may represent a marker of more severe EP impairment. Other factors such as medication use, or age may confound differences between illness stages. While findings regarding the effects of antipsychotics on EP are equivocal,^97,98^ it is likely that individuals with greater positive symptom severity (as in established psychosis) are taking higher antipsychotic medication doses, which may further impact EP performance. A recent study found that EP performance was worse in older individuals with psychosis than in younger age groups,^99^ although age did not moderate the strength of the relationship between EP and symptoms across stages. Antipsychotic dose or age did not moderate any of the examined relationships in this review, however, the power to detect moderating effects was limited, particularly by the small number of studies reporting medication use. Further examining the effects of medication and age on these relationships may therefore represent an avenue for further research. Nevertheless, the current findings regarding the illness stage should be interpreted carefully, given the small number of included FEP studies.

### Moderating Effects of EP Task Type

The stronger estimated relationship between negative symptoms and higher-level EP compared with lower-level EP tasks is another novel, and potentially important, finding. It has been suggested that impaired reward-related learning processes could be implicated in emotion regulation and management abilities, as well as motivational aspects of negative symptoms (eg, anhedonia, asociality).^100^ If so, this shared underlying mechanism could partially explain the closer links between negative symptoms with higher-level EP observed here. There were no other significant differences between lower-level and higher-level EP tasks. The estimated correlation with social functioning was slightly larger for higher-level tasks, however, and future studies may determine whether tasks tapping into higher- versus lower- social order cognitive processes are more sensitive to social functioning impairment. While the underlying factor structure of social cognition remains unclear,^2^ findings from this review suggest that considering lower- and higher-level processing separately might better help us understand the relationships between symptoms and social functioning.

### Clinical Implications

Social cognitive training (SCT) programs developed over the past decade have shown some promise for improving social cognition and social functioning in people with psychosis.^8,101^ However, they are yet to consistently lead to improvements in social functioning.^101^ The current review found only a small relationship between EP social functioning, and a smaller indirect relationship from EP to social functioning mediated by symptoms, suggesting that there is a limit to the potential for symptom reduction and improvement in social functioning that can be expected through remediation of EP. While EP represents only one domain of cognition known to be related to social functioning in psychotic disorders,^11,13^ the larger correlations between the symptom domains and social functioning suggest that interventions combining SCT with those targeting amelioration of negative and disorganization symptoms, may provide the greatest effect on social functioning. Relationships between EP, symptoms, and social functioning may, in fact, be bidirectional, and personalized approaches that consider the interplay between these constructs for the individual, and incorporate a range of interventions such as SCT, psychosocial interventions, and a focus on symptoms may be necessary to improve social functioning. Such interventions may require care across multidisciplinary teams or services.

The review provides some preliminary evidence that the strength of the relationship between EP and social functioning may vary across early (ie, FEP) and established stages of illness, though more research is clearly needed in individuals with FEP to investigate this further. The findings tentatively suggest that early intervention to improve EP in the early stages of illness may be warranted, and social cognitive remediation programs do appear to be similarly effective for individuals with FEP and those with chronic psychosis.^102^ A better understanding of how EP is related to symptoms and social functioning at the onset of psychotic illness may provide clues for how to prevent patterns of EP deficits, symptom severity, and social dysfunction from becoming entrenched and protect against deterioration throughout the disorder.

### Limitations

There are some limitations worth noting. First, there was a small number of included FEP studies, and we were unable to examine the moderating effects of the stage of illness on correlations involving depressive and disorganization symptoms. Second, most included studies were conducted on exclusively non-affective psychosis cohorts. Individuals with affective psychoses typically experience social cognitive impairments,^103^ somewhat beyond individuals with mood disorders without psychosis,^104^ and therefore represent an understudied population in this area. Third, there was substantial between-study heterogeneity, particularly in symptom-social functioning relationships, that was not explained by moderators. This may be partly related to the wide variety of measurement tools used, highlighting the importance of consensus and consistency in measurement across all constructs examined here. While recent work has sought to reach a consensus on the best measures of social cognition,^89^ further work exploring the psychometric properties of social functioning measures is needed.^37^ Studies have been increasingly conceptualizing symptoms using 5-factor classifications (ie, positive, negative, disorganization, depression, and excited factors) and the current finding that removing disorganization items reduced the correlation between EP and positive symptoms supports this approach. Fourth, as most included studies reported total symptom domain scores and total social functioning scores, our exploratory analysis of negative symptom subdomains was limited. Further research that includes, for example, relationships between individual symptoms and subdomains of social functioning (eg, relationships and productive activities) is warranted.

While we used a staging approach to assess the moderating effects of the illness stage, many included studies did not differentiate between individuals experiencing early or chronic stages of the disorder. Consequently, some cohorts we classified as established psychosis might have included individuals with both early and late-stage disorders. While the long average duration of illness reported in most established psychosis cohorts suggests that our approach was appropriate, subgroup analyses comparing relationships across stages of illness should still be interpreted cautiously as, together with the small number of FEP cohorts, the potential mixture of stages may have limited the ability to detect differences. More research is needed to directly compare these relationships across different stages of psychosis (eg, as in Brasso et al.^49^). Finally, excluding non-English language studies may limit the cross-cultural generalizability of findings, and including only cross-sectional associations limits our ability to consider causal explanations, though mediation analyses can provide some evidence for plausible models of relationships.

## Conclusions

Findings from this systematic review and meta-analysis have provided evidence that symptoms, particularly negative and disorganization symptoms, partially mediate the relationship between EP and social functioning in psychosis, though the indirect effects through symptoms were small. The study provided novel findings that higher-level EP tasks were more strongly associated with negative symptoms than lower-level EP tasks, and exploring this further may provide insights into the mechanisms underlying their relationship. The findings suggest that a holistic approach to the treatment of psychosis that targets both social cognition and symptoms may be the most effective way to improve social functioning and highlight that further research is needed to more conclusively determine whether different interventions may be needed for individuals at early or established stages of illness.

## Supplementary Material

Supplementary material is available at https://academic.oup.com/schizophreniabulletin/.

sbae167_suppl_Supplementary_Material
