# Supplementary material for: Emotion Processing and Its Relationship to Social Functioning and Symptoms in Psychotic Disorder: A Systematic Review and Meta-analysis
Source: Schizophr Bull. 2024 Sep 4;51(4):1054–71. doi: 10.1093/schbul/sbae167 (PMC12236351; doi:10.1093/schbul/sbae167)
Supplement: sbae167_suppl_Supplementary_Material [file sbae167_suppl_supplementary_material.docx]

**Supplementary Materials**

**Emotion Processing and its Relationship to Social Functioning and Symptoms in Psychotic Disorder: A Systematic Review and Meta-analysis**

Sean Murrihy, Kate Filia, Sue Cotton, Lisa Phillips, Sarah Youn, Anuradhi Jayasinghe, Anna Wrobel, Eslam M. Bastawy, Kelly Allott, Amity Watson

**Table S1.** PRISMA Checklist

**Search Strategy**

**Table S2.** PsycInfo Search Strategy

**Table S3.** Embase Search Strategy

**Table S4.** Medline Search Strategy

**Table S5.** List of Data Extracted from Each Study

**Quality Appraisal**

**Table S6.** Quality Appraisal Checklist

**Table S7.** Excluded Studies Close to Meeting Inclusion Criteria and the Reason for Exclusion

**Table S8.** Emotion Processing Measures Used in the Included Studies

**Table S9.** Symptom Measures Used in the Included Studies

**Table S10.** Social Functioning Measures Used in the Included Studies

**Meta Analyses Results**

**Fig. S1 – S18.** Forest Plots and Funnel Plots

**Table S11**. Moderator Analysis (Meta-Regression) for the Nine Outcome Correlation Pairs

**Fig. S19.** Mediation analyses

**Table S12.** Exploratory Meta-analyses for the Two Subdomains of Negative Symptoms

**Sensitivity Analysis**

**Table S13.** Sensitivity Analyses Results

**Table S1.** PRISMA Checklist

| **Section and Topic** | **Item #** | **Checklist item** | **Location where item is reported** |
| --- | --- | --- | --- |
| **TITLE** | | |  |
| Title | 1 | Identify the report as a systematic review. | 1 |
| **ABSTRACT** | | |  |
| Abstract | 2 | See the PRISMA 2020 for Abstracts checklist. | 2 |
| **INTRODUCTION** | | |  |
| Rationale | 3 | Describe the rationale for the review in the context of existing knowledge. | 3-5 |
| Objectives | 4 | Provide an explicit statement of the objective(s) or question(s) the review addresses. | 5 |
| **METHODS** | | |  |
| Eligibility criteria | 5 | Specify the inclusion and exclusion criteria for the review and how studies were grouped for the syntheses. | 6 |
| Information sources | 6 | Specify all databases, registers, websites, organisations, reference lists and other sources searched or consulted to identify studies. Specify the date when each source was last searched or consulted. | 5 |
| Search strategy | 7 | Present the full search strategies for all databases, registers and websites, including any filters and limits used. | Supplementary Tables S2:S4 |
| Selection process | 8 | Specify the methods used to decide whether a study met the inclusion criteria of the review, including how many reviewers screened each record and each report retrieved, whether they worked independently, and if applicable, details of automation tools used in the process. | 8 |
| Data collection process | 9 | Specify the methods used to collect data from reports, including how many reviewers collected data from each report, whether they worked independently, any processes for obtaining or confirming data from study investigators, and if applicable, details of automation tools used in the process. | 8-9 |
| Data items | 10a | List and define all outcomes for which data were sought. Specify whether all results that were compatible with each outcome domain in each study were sought (e.g. for all measures, time points, analyses), and if not, the methods used to decide which results to collect. | 6-9 |
|  | 10b | List and define all other variables for which data were sought (e.g. participant and intervention characteristics, funding sources). Describe any assumptions made about any missing or unclear information. | 8-9; Supplementary Table S5 |
| Study risk of bias assessment | 11 | Specify the methods used to assess risk of bias in the included studies, including details of the tool(s) used, how many reviewers assessed each study and whether they worked independently, and if applicable, details of automation tools used in the process. | 9; Supplementary Table S6 |
| Effect measures | 12 | Specify for each outcome the effect measure(s) (e.g. risk ratio, mean difference) used in the synthesis or presentation of results. | 9 |
| Synthesis methods | 13a | Describe the processes used to decide which studies were eligible for each synthesis (e.g. tabulating the study intervention characteristics and comparing against the planned groups for each synthesis (item #5)). | 9-10 |
|  | 13b | Describe any methods required to prepare the data for presentation or synthesis, such as handling of missing summary statistics, or data conversions. | 9-10 |
|  | 13c | Describe any methods used to tabulate or visually display results of individual studies and syntheses. | NA |
|  | 13d | Describe any methods used to synthesize results and provide a rationale for the choice(s). If meta-analysis was performed, describe the model(s), method(s) to identify the presence and extent of statistical heterogeneity, and software package(s) used. | 9-10 |
|  | 13e | Describe any methods used to explore possible causes of heterogeneity among study results (e.g. subgroup analysis, meta-regression). | 10 |
|  | 13f | Describe any sensitivity analyses conducted to assess robustness of the synthesized results. | 10; Supplementary Materials |
| Reporting bias assessment | 14 | Describe any methods used to assess risk of bias due to missing results in a synthesis (arising from reporting biases). | NA |
| Certainty assessment | 15 | Describe any methods used to assess certainty (or confidence) in the body of evidence for an outcome. | NA |
| **RESULTS** | | |  |
| Study selection | 16a | Describe the results of the search and selection process, from the number of records identified in the search to the number of studies included in the review, ideally using a flow diagram. | 11; Figure 1 |
|  | 16b | Cite studies that might appear to meet the inclusion criteria, but which were excluded, and explain why they were excluded. | 11; Supplementary Table S7 |
| Study characteristics | 17 | Cite each included study and present its characteristics. | Table 1 |
| Risk of bias in studies | 18 | Present assessments of risk of bias for each included study. | Table 1 |
| Results of individual studies | 19 | For all outcomes, present, for each study: (a) summary statistics for each group (where appropriate) and (b) an effect estimate and its precision (e.g. confidence/credible interval), ideally using structured tables or plots. | Supplementary Figures S1—S15 |
| Results of syntheses | 20a | For each synthesis, briefly summarise the characteristics and risk of bias among contributing studies. | 1; Table 1 |
|  | 20b | Present results of all statistical syntheses conducted. If meta-analysis was done, present for each the summary estimate and its precision (e.g. confidence/credible interval) and measures of statistical heterogeneity. If comparing groups, describe the direction of the effect. | 12-15 |
|  | 20c | Present results of all investigations of possible causes of heterogeneity among study results. | 13-15; Supplementary Table S11 |
|  | 20d | Present results of all sensitivity analyses conducted to assess the robustness of the synthesized results. | 14-15; Supplementary Table S13 |
| Reporting biases | 21 | Present assessments of risk of bias due to missing results (arising from reporting biases) for each synthesis assessed. | NA |
| Certainty of evidence | 22 | Present assessments of certainty (or confidence) in the body of evidence for each outcome assessed. | NA |
| **DISCUSSION** | | |  |
| Discussion | 23a | Provide a general interpretation of the results in the context of other evidence. | 16-20 |
|  | 23b | Discuss any limitations of the evidence included in the review. | 20-21 |
|  | 23c | Discuss any limitations of the review processes used. | 21 |
|  | 23d | Discuss implications of the results for practice, policy, and future research. | 19-22 |
| **OTHER INFORMATION** | | |  |
| Registration and protocol | 24a | Provide registration information for the review, including register name and registration number, or state that the review was not registered. | 5 |
|  | 24b | Indicate where the review protocol can be accessed, or state that a protocol was not prepared. | 5 |
|  | 24c | Describe and explain any amendments to information provided at registration or in the protocol. | NA |
| Support | 25 | Describe sources of financial or non-financial support for the review, and the role of the funders or sponsors in the review. | 31 |
| Competing interests | 26 | Declare any competing interests of review authors. | 31 |
| Availability of data, code and other materials | 27 | Report which of the following are publicly available and where they can be found: template data collection forms; data extracted from included studies; data used for all analyses; analytic code; any other materials used in the review. | NA |

*Note:* Table taken from Page et al.^1^

**Table S2.** PsycInfo Search Strategy 5 September 2023

|  | **Search terms** | **Retrievals** |
| --- | --- | --- |
| 1 | exp psychosis/ or exp schizophrenia/ or exp bipolar disorder/ or exp schizoaffective disorder/ | 156789 |
| 2 | (Psychosis or psychoses or psychotic or schizophren* or schizoaffective or delusional disorder*).ti,ab. | 181431 |
| 3 | (Manic depression or bipolar disorder* or bipolar affective disorder* or bipolar depression).ti,ab. | 33654 |
| 4 | (risk mental state or clinical high risk or ultra high risk).ti,ab. | 2447 |
| 5 | 1 or 2 or 3 or 4 | 216375 |
| 6 | exp "Theory of Mind"/ | 7022 |
| 7 | exp social cognition/ or exp mentalization/ or exp emotion recognition/ | 23257 |
| 8 | Social cognit*.ti,ab. | 23258 |
| 9 | (social knowledge or social perception or social judgment or social cue*).ti,ab. | 6965 |
| 10 | (mentalising or mentalizing or mentalisation or mentalization or theory of mind or perspective taking or mind reading).ti,ab. | 17135 |
| 11 | (Emotion perception or face perception or affect perception or emotion identification or affect recognition or emotion recognition or emotion processing or face processing or affect processing or prosody).ti,ab. | 15657 |
| 12 | attribution*.ti,ab. | 31110 |
| 13 | 6 or 7 or 8 or 9 or 10 or 11 or 12 | 95939 |
| 14 | exp "Positive and Negative Symptoms"/ | 4125 |
| 15 | exp "Depression (Emotion)"/ | 27014 |
| 16 | exp hallucinations/ or exp delusions/ or exp anhedonia/ or exp apathy/ | 15895 |
| 17 | (depress* adj symptom*).ti,ab. | 66616 |
| 18 | (positive symptom* or delusion* or hallucinat* or thought disorder* or psychotic symptom*).ti,ab. | 41164 |
| 19 | (negative symptom* or anhedonia or amotivation or alogia or asociality or apathy or avolition or blunted affect or restricted affect or social withdrawal or (affect* adj flat*)).ti,ab. | 23129 |
| 20 | 14 or 15 or 16 or 17 or 18 or 19 | 146964 |
| 21 | exp "Activities of Daily Living"/ | 6953 |
| 22 | exp social adjustment/ or exp social exclusion/ or exp social functioning/ or exp social inclusion/ or exp social skills/ or exp employment status/ or exp educational attainment level/ | 70869 |
| 23 | (Functional capacity or functional outcome* or social function* or social dysfunction).ti,ab. | 23813 |
| 24 | (Social behaviour or social behavior or social adjustment or social competence or social skills).ti,ab. | 43342 |
| 25 | (Daily functioning or activities of daily living or living skills).ti,ab. | 14376 |
| 26 | (Vocational outcome* or employment or unemployment or occupational functioning or vocational functioning or neet).ti,ab. | 68687 |
| 27 | (academic functioning or school completion or school attendance or educational status).ti,ab. | 5215 |
| 28 | (Community functioning or community behaviour or community behavior).ti,ab. | 514 |
| 29 | 21 or 22 or 23 or 24 or 25 or 26 or 27 or 28 | 186624 |
| 30 | 20 or 29 | 324335 |
| 31 | 5 and 13 and 30 | 2297 |
| 32 | books/ or case report/ or meta analysis/ or literature review/ or (case report or case series or systematic review or meta-analysis or literature review or conference abstract or conference review).ti,ab. or (book or authored book or edited book or dissertation abstract or encyclopedia or non-peer-reviewed journal).pt. | 1243196 |
| 33 | 31 not 32 | 1940 |
| 34 | (afrikaans or albanian or arabic or bulgarian or catalan or chinese or czech or danish or dutch or finnish or french or georgian or german or greek or hebrew or hindi or hungarian or iranian or italian or japanese or korean or lithuanian or malaysian or nonenglish or norwegian or polish or portuguese or romanian or russian or serbo croatian or slovak or slovene or spanish or swedish or turkish or ukrainian).lg. | 333746 |
| 35 | 33 not 34 | 1792 |
| 36 | (animal not human).po. | 386913 |
| 37 | 35 not 36 | 1771 |
| 38 | limit 37 to yr="1990 -Current" | 1744 |
|  |  |  |

**Table S3.** Embase Search Strategy 5 September 2023

|  |  | **Search terms** | **Retrievals** |  |
| --- | --- | --- | --- | --- |
|  | 1 | exp schizoaffective psychosis/ or exp psychosis/ or exp schizophrenia spectrum disorder/ or exp bipolar depression/ or exp bipolar disorder/ | 409198 |  |
|  | 2 | (psychosis or psychoses or psychotic or schizophren* or schizoaffective or delusional disorder*).ti,ab. | 275235 |  |
|  | 3 | (Manic depression or bipolar disorder* or bipolar affective disorder* or bipolar depression).ti,ab. | 58359 |  |
|  | 4 | (risk mental state or clinical high risk or ultra high risk).ti,ab. | 5387 |  |
|  | 5 | 1 or 2 or 3 or 4 | 448612 |  |
|  | 6 | exp "theory of mind"/ | 5912 |  |
|  | 7 | exp social cognition/ or mentalization/ or exp facial recognition/ or exp voice recognition/ | 16855 |  |
|  | 8 | exp "attribution (psychology)"/ | 1424 |  |
|  | 9 | social cognit*.ti,ab. | 16991 |  |
|  | 10 | (mentalising or mentalizing or mentalisation or mentalization or theory of mind or perspective taking or mind reading).ti,ab. | 11761 |  |
|  | 11 | (Emotion perception or face perception or affect perception or emotion identification or affect recognition or emotion recognition or emotion processing or face processing or affect processing or prosody).ti,ab. | 18311 |  |
|  | 12 | attribution*.ti,ab. | 22002 |  |
|  | 13 | (social knowledge or social perception or social judgment or social cue).ti,ab. | 2532 |  |
|  | 14 | 6 or 7 or 8 or 9 or 10 or 11 or 12 or 13 | 73210 |  |
|  | 15 | exp positive syndrome/ or exp hallucination/ or exp delusion/ or exp negative symptoms/ or exp anhedonia/ | 94202 |  |
|  | 16 | (depress* adj symptom*).ti,ab. | 103753 |  |
|  | 17 | (positive symptom* or delusion* or hallucinat* or thought disorder*).ti,ab. | 51406 |  |
|  | 18 | (negative symptom* or anhedonia or amotivation or alogia or asociality or apathy or avolition or blunted affect or restricted affect or social withdrawal or (affect* adj flat*)).ti,ab. | 38664 |  |
|  | 19 | 15 or 16 or 17 or 18 | 225149 |  |
|  | 20 | exp daily life activity/ or exp social adaptation/ or exp social competence/ or exp social disability/ or exp social inclusion/ or exp employment status/ or exp academic achievement/ | 365090 |  |
|  | 21 | (Functional capacity or functional outcome* or social function* or social dysfunction).ti,ab. | 139183 |  |
|  | 22 | (Social behaviour or social behavior or social adjustment or social competence or social skills).ti,ab. | 30261 |  |
|  | 23 | (Daily functioning or activities of daily living or living skills).ti,ab. | 53709 |  |
|  | 24 | (Vocational outcome* or employment or unemployment or occupational functioning or vocational functioning or neet).ti,ab. | 117023 |  |
|  | 25 | (academic functioning or school completion or school attendance or educational status).ti,ab. | 10329 |  |
|  | 26 | (Community functioning or community behaviour or community behavior).ti,ab. | 837 |  |
|  | 27 | 20 or 21 or 22 or 23 or 24 or 25 or 26 | 620899 |  |
|  | 28 | 19 or 27 | 825060 |  |
|  | 29 | 5 and 14 and 28 | 4430 |  |
|  | 30 | book/ or case report/ or meta analysis/ or review/ or (case report or case series or systematic review or meta-analysis or literature review or conference abstract or conference paper or conference review).ti,ab. or (books or chapter or conference abstract or conference paper or conference review or editorial or letter or note or review or short survey).pt. | 14960586 |  |
|  | 31 | 29 not 30 | 2314 |  |
|  | 32 | (afrikaans or albanian or arabic or bulgarian or catalan or chinese or czech or danish or dutch or finnish or french or georgian or german or greek or hebrew or hindi or hungarian or iranian or italian or japanese or korean or lithuanian or malaysian or nonenglish or norwegian or polish or portuguese or romanian or russian or serbo croatian or slovak or slovene or spanish or swedish or turkish or ukrainian).lg. | 6415453 |  |
|  | 33 | 31 not 32 | 2193 |  |
|  | 34 | exp animal/ not human/ | 6085596 |  |
|  | 35 | 33 not 34 | 2166 |  |
|  | 36 | limit 35 to yr="1990 -Current" | 2144 |  |

**Table S4.** Medline Search Strategy 5 September 2023

|  |  | **Search terms** | **Retrievals** |  |
| --- | --- | --- | --- | --- |
|  | 1 | exp psychotic disorders/ or exp Schizophrenia, Catatonic/ or exp Schizophrenia, Disorganized/ or exp Schizophrenia/ or exp Schizophrenia, Paranoid/ or exp Schizophrenia, Childhood/ or exp Affective Disorders, Psychotic/ or exp Bipolar Disorder/ | 194931 |  |
|  | 2 | (psychosis or psychoses or psychotic or schizophren* or schizoaffective or delusional disorder*).ti,ab. | 189653 |  |
|  | 3 | (Manic depression or bipolar disorder* or bipolar affective disorder* or bipolar depression).ti,ab. | 37842 |  |
|  | 4 | (risk mental state or clinical high risk or ultra high risk).ti,ab. | 2945 |  |
|  | 5 | 1 or 2 or 3 or 4 | 264686 |  |
|  | 6 | exp "Theory of Mind"/ | 3713 |  |
|  | 7 | exp mentalization/ or exp facial recognition/ or exp voice recognition/ or exp social perception/ | 29474 |  |
|  | 8 | Social cognit*.ti,ab. | 13606 |  |
|  | 9 | (mentalising or mentalizing or mentalisation or mentalization or theory of mind or perspective taking or mind reading).ti,ab. | 9709 |  |
|  | 10 | (Emotion perception or face perception or affect perception or emotion identification or affect recognition or emotion recognition or emotion processing or face processing or affect processing or prosody).ti,ab. | 15041 |  |
|  | 11 | (social knowledge or social perception or social judgment or social cue).ti,ab. | 2210 |  |
|  | 12 | attribution*.ti,ab. | 15615 |  |
|  | 13 | 6 or 7 or 8 or 9 or 10 or 11 or 12 | 73262 |  |
|  | 14 | exp affective symptoms/ or exp delusions/ or paranoid behavior/ or schizophrenic language/ or hallucinations/ or anhedonia/ or exp Apathy/ | 35670 |  |
|  | 15 | (depress* adj symptom*).ti,ab. | 79737 |  |
|  | 16 | (positive symptom* or delusion* or hallucinat* or thought disorder*).ti,ab. | 31468 |  |
|  | 17 | (negative symptom* or anhedonia or amotivation or alogia or asociality or apathy or avolition or blunted affect or restricted affect or social withdrawal or (affect* adj flat*)).ti,ab. | 24737 |  |
|  | 18 | 14 or 15 or 16 or 17 | 149353 |  |
|  | 19 | exp "Activities of Daily Living"/ | 120763 |  |
|  | 20 | exp social adjustment/ or exp social inclusion/ or exp social isolation/ or exp social skills/ or exp psychosocial functioning/ or exp employment/ or exp educational status/ | 205582 |  |
|  | 21 | (Functional capacity or functional outcome* or social function* or social dysfunction or psychosocial function*).ti,ab. | 98967 |  |
|  | 22 | (Social behaviour or social behavior or social adjustment or social competence or social skills).ti,ab. | 23919 |  |
|  | 23 | (Daily functioning or activities of daily living or living skills).ti,ab. | 37855 |  |
|  | 24 | (Vocational outcome* or work functioning or employment or unemployment or occupational functioning or vocational functioning or neet).ti,ab. | 83875 |  |
|  | 25 | (academic functioning or school completion or school attendance or educational status).ti,ab. | 7779 |  |
|  | 26 | (Community functioning or community behaviour or community behavior).ti,ab. | 624 |  |
|  | 27 | 19 or 20 or 21 or 22 or 23 or 24 or 25 or 26 | 505525 |  |
|  | 28 | 18 or 27 | 638185 |  |
|  | 29 | 5 and 13 and 28 | 2358 |  |
|  | 30 | books/ or case reports/ or meta analysis/ or review/ or systematic review/ or (case report or case series or systematic review or meta-analysis or literature review or conference abstract or conference review).ti,ab. or (case reports or comment or editorial or letter or meta analysis or news or review or systematic review).pt. | 7918049 |  |
|  | 31 | 29 not 30 | 1940 |  |
|  | 32 | (afrikaans or albanian or arabic or armenian or azerbaijani or belorussian or bengali or bosnian or bulgarian or burmese or catalan or chinese or croatian or czech or danish or dutch or esperanto or estonian or finnish or flemish or french or gaelic, scots or georgian or german or greek or hausa or hebrew or hindi or hungarian or icelandic or indonesian or interlingua or italian or japanese or kirghiz or korean or latin or latvian or lithuanian or macedonian or malay or marathi or masai or multilingual or norwegian or persian or polish or portuguese or pushto or rumanian or russian or serbian or slovak or slovene or spanish or swahili or swedish or tagalog or tamil or telugu or thai or turkish or ukrainian or undetermined or urdu or vietnamese or welsh).lg. | 4908716 |  |
|  | 33 | 31 not 32 | 1866 |  |
|  | 34 | exp animals/ not humans/ | 5151321 |  |
|  | 35 | 33 not 34 | 1846 |  |
|  | 36 | limit 35 to yr="1990 -Current" | 1811 |  |

**Table S5.** List of Data Extracted from Each Study

| **Study component** | **Data extracted** |
| --- | --- |
| Study characteristics | Country, setting (i.e., inpatient, outpatient), inclusion criteria, diagnostic criteria used, method of diagnosis |
| Cohort characteristics | Number of participants, age (mean, SD), gender (% male), diagnoses of participants, duration of illness (mean, SD), antipsychotic dose - CPZ equivalent (mean, SD) |
| Details of outcome measures | Emotion processing tests used, emotion processing test performance (mean, SD), symptom measures used, symptom severity (mean, SD), social functioning tests used, social functioning performance (mean, SD) |
| Effects | Correlation effect size, confidence interval, p value, number of participants for correlation were extracted for each correlation between: EP measures and symptom measures, EP measures and social functioning measures, symptom measures and social functioning |
| *Note:* SD, standard deviation; CPZ, chlorpromazine |  |
|  |  |

**Quality appraisal**

The following table, adapted from the Joanna Briggs Institute (JBI) critical appraisal checklist for Analytical Cross-Sectional Studies,^2^ was used for methodological quality appraisal. Due to inconsistency in the literature surrounding confounds identified as important for the examined relationships and the fact that bivariate correlations were extracted, items related to confounds on the JBI checklist were omitted on the adapted checklist. Responses of ‘yes’ were given a score of 1 and responses of ‘no’ or ‘unclear’ were both scored 0. This provided a quality score out of 7 for each study.

**Table S6.** Quality Appraisal Checklist

| **Criteria and description** | **Responses** |
| --- | --- |
| **1. Were the criteria for inclusion in the sample clearly defined?**  The authors should provide clear inclusion and exclusion criteria that they developed prior to recruitment of the study participants. The inclusion/exclusion criteria should be specified with sufficient detail and all the necessary information critical to the study. | Yes / No / Unclear |
| **2. Were the study subjects and the setting described in detail?**  The study sample should be described in sufficient detail so that other researchers can determine if it is comparable to the population of interest to them. The authors should provide a clear description of the population from which the study participants were selected or recruited, including demographics, and location. To meet criteria, studies must report setting description, age, and gender, and additionally report three other demographic variables (e.g., level of education, ethnicity, illness duration, age of onset, medication, IQ). | Yes / No / Unclear |
| **3. Were objective, standard criteria used for measurement of the condition?**  Diagnosis should be made according to recognised diagnostic criteria (e.g., DSM, ICD) with a validated measure (e.g., SCID, MINI); if inclusion to study was based on psychiatrist assessment, this should be confirmed with a validated measure. | Yes / No / Unclear |
| **4. Were the social cognition outcomes measured in a valid and reliable way?**  Is the social cognition outcome assessed based on existing recognised definitions of the social cognitive domain? Was social cognition measured with a recognised, validated tool? Further, was scoring done in a recognised, validated way? | Yes / No / Unclear |
| **5. Were the clinical symptom outcomes clinical symptoms measured in a valid and reliable way?**  Were validated tools (e.g., PANSS, SAPS) used to measure symptoms? Further, were these tools used appropriately for measurement of symptoms – for example, if alternative scoring systems are used for symptom assessment (e.g., 5-factor scoring of PANSS), have these been previously validated? | Yes / No / Unclear |
| **6. Were the social functioning outcomes measured in a valid and reliable way?**  Was social functioning measured with a validated tool? Were these used/scored appropriately? If informant ratings were used, were these appropriate? | Yes / No / Unclear |
| **7. Was appropriate statistical analysis used?**  Did they report associations appropriately, clearly enough to identify which analytical techniques were used, whether assumptions associated with approach reasonable, and whether any confounders were measured/analysed? | Yes / No / Unclear |

**Table S7.** Excluded Studies Close to Meeting Inclusion Criteria and the Reason for Exclusion

| **Study** | **Exclusion Reason** |
| --- | --- |
| Smith et al.^3^ | This study met inclusion criteria, however the cohort appeared to be overlapping with the cohort included in Abram et al.^4^ While both studies included similar cohort size, data was extracted from Abram et al. as it provided data for a greater number of social skills tasks (not included in this analysis). |
| Martinez-Dominguez et al.^5^ | This study appeared to meet inclusion criteria and provided correlations between all three outcomes of interest, however it was unclear whether the reported correlations were ascertained from individuals with psychosis only, or the entire sample which also included healthy controls. Authors did not respond to a request for clarification; therefore, it was decided to exclude the study. |
| Green et al.^6^ | This study reported correlations between the three outcomes of interest, however it was unclear from the methodology whether these represented zero-order bivariate correlations or partial correlations estimated from the covariance matrix. Authors did not respond to a request for clarification; therefore, it was decided to exclude the study. |

**Table S8.** Emotion Processing Measures Used in the Included Studies

| **Emotion Processing Tasks** | **Studies** |
| --- | --- |
| ***Lower-level Processing*** |  |
| Facial Affect Identification Task (FEIT)^7^ | 6 |
| Bell Lysaker Emotion Recognition Test (BLERT)^8^ | 5 |
| Penn Emotion Recognition Task (ER-40)^9^ | 4 |
| Facial Affect Discrimination Task (FEDT)^7^ | 3 |
| Emotion in Biological Motion Point-light Task^10^ | 2 |
| Facial Expression of Emotion: Stimulus and Test (FEEST)^11^ | 2 |
| Body Emotion Recognition Task (BR-100)^12^ | 1 |
| Cambridge Neuropsychological Test Automated Battery (CANTAB) Emotion Recognition Task^13^ | 1 |
| Chinese Facial Emotion Recognition Database (CFERD)^14^ | 1 |
| Face-Affective Identification Task - Chinese Version (C-FAIT)^15^ | 1 |
| Emotion recognition task^16^ | 1 |
| Faces test^17^ | 1 |
| Facial affect perception (FAP) task^18^ | 1 |
| Facial affect recognition (FAR) task^19^ | 1 |
| Frankfurt Test and Training of Facial Affect Recognition 2nd Edition (FEFA-2)^20^ | 1 |
| Pictures of Facial Affect (POFA)^21^ | 1 |
| Penn Emotion Discrimination Task^22^ | 1 |
| Prosody task ^23^ | 1 |
| Tool for Recognition of Emotions in Neuropsychiatric Disorders (TRENDS)^24^ | 1 |
| ***Higher Level Processing*** |  |
| Mayer-Salovey-Caruso Emotional Intelligence Test - Managing Emotions (MSCEIT-ME)^25^ | 10^a^ |
| Toronto Alexithymia Scale (TAS)^26^ | 2 |
| Trait Meta-Mood Scale (TMMS)^27^ | 1 |
| ^a^ MSCEIT-ME was used in 10 independent studies, one which included two cohorts at different stages of illness, resulting in 11 cohorts included in the analysis using this measure.  Note that as several studies used multiple measures, the summed total of the studies column is greater than the number of included studies. | |

**Table S9.** Symptom Measures Used in the Included Studies

| **Symptom Measures** | **Studies** |
| --- | --- |
| ***Positive symptoms*** |  |
| Positive and Negative Syndrome Scale (PANSS)^28^ – Positive subscale score | 12 |
| Positive and Negative Syndrome Scale (PANSS)^28^ – 5 factor solution score | 9^a^ |
| Scale for the Assessment of Positive Symptoms (SAPS)^29^ – Total scale score | 4 |
| Brief Psychiatric Rating Scale (BPRS)^30^ Positive subscale score | 2 |
| Brief Psychiatric Rating Scale (BPRS)^30^ – Positive symptom factor solution score | 2 |
| Comprehensive Assessment of At Risk Mental States (CAARMS)^31^ | 1 |
| Clinical Global Impression – Schizophrenia (CGI-S)^32^ | 1 |
| Scale for the Assessment of Positive Symptoms (SAPS)^29^ – Hallucinations and delusion items | 1 |
| ***Negative symptoms*** |  |
| Positive and Negative Syndrome Scale (PANSS)^28^ – Negative subscale score | 11 |
| Positive and Negative Syndrome Scale (PANSS)^28^ – 5 factor solution score | 7 |
| Scale for the Assessment of Negative Symptoms (SANS)^29^ – Total scale score | 3 |
| Brief Negative Symptoms Scale (BNSS)^33^ | 2^a^ |
| Brief Psychiatric Rating Scale (BPRS)^30^ – Negative symptom factor solution score | 2 |
| Scale for the Assessment of Negative Symptoms (SANS)^29^ – Total scale score minus attention item | 2 |
| Brief Psychiatric Rating Scale (BPRS)^30^ Negative subscale score | 1 |
| Clinical Assessment Interview for Negative Symptoms (CAINS)^34^ | 1 |
| Clinical Global Impression – Schizophrenia (CGI-S)^32^ | 1 |
| Scale for the Assessment of Negative Symptoms (SANS)^29^ – Total scale score minus attention and inappropriate affect items | 1 |
| ***Disorganisation Symptoms*** |  |
| Positive and Negative Syndrome Scale (PANSS)^28^ – 5 factor solution score | 7^a^ |
| Brief Psychiatric Rating Scale (BPRS)^30^ – Disorganisation symptom factor solution score | 1 |
| Clinical Global Impression – Schizophrenia (CGI-S)^32^ | 1 |
| Positive and Negative Syndrome Scale (PANSS) – Conceptual disorganisation item | 1 |
| Sum of SAPS^29^ positive formal thought disorder and bizarre behavior items and SANS^29^ attention item | 1 |
| ***Depressive symptoms*** |  |
| Calgary Depression Scale for Schizophrenia (CDSS)^35^ | 5^a^ |
| Positive and Negative Syndrome Scale (PANSS)^28^ – 5 factor solution score | 5 |
| Beck Depression Inventory (BDI-II) ^36^ | 3 |
| Hamilton Depression Rating Scale (HAMD)^37^ | 3 |
| Clinical Global Impression – Schizophrenia (CGI-S)^32^ | 1 |
| ^a^ These measures were used in one study which included two cohorts at different stages of illness, so the number of cohorts included in the meta-analysis using these measures equals the number of studies plus one.  Note that as several studies used multiple measures, the summed total of the studies column is greater than the number of included studies. | |

**Table S10.** Social Functioning Measures Used in the Included Studies

| **Social Functioning Measure** | **Studies** |
| --- | --- |
| The Social Functioning Scale (SFS)^38^ | 10 |
| The Personal and Social Performance Scale (PSP)^39^ | 8 |
| Global Assessment of Functioning (GAF)^40^ | 5 |
| Specific levels of functioning scale (SLOF)^41^ | 3^a^ |
| Quality of Life Scale (QLS)^42^ | 3 |
| Social Adjustment Scale – Self Report (SAS-SR) ^43^ | 2 |
| Role functioning scale (RFS)^44^ | 2 |
| Groningen Social Disabilities Schedule (GSDS)^45^ | 1 |
| Independent Living Scales Survey (ILSS)^46^ | 1 |
| Quality of Life Scale (QLS)^42^ – Interpersonal Relations subscale | 1 |
| Social Adjustment Scale Interview (SAS-II)^47^ | 1 |
| Specific Levels of Functioning Scale (SLOF)^41^ – Sum of Interpersonal Relationships and Social Appropriateness Subscales | 1 |
| Social and Occupational Functioning Assessment Scale (SOFAS)^48^ | 1 |
| Specific Levels of Functioning Scale (SLOF)^41^ – Interpersonal Relationships subscale | 1 |
| World Health Organisation Disability Assessment Schedule (WHODAS) 2.0^49^ | 1 |
| ^a^ SLOF was used in 3 independent studies, one which included two cohorts at different stages of illness, resulting in 4 cohorts included in the analysis using this measure. Note that as several studies used multiple measures, the summed total of the studies column is greater than the number of included studies. | |

**Meta Analyses Results: Forest Plots and Funnel Plots**


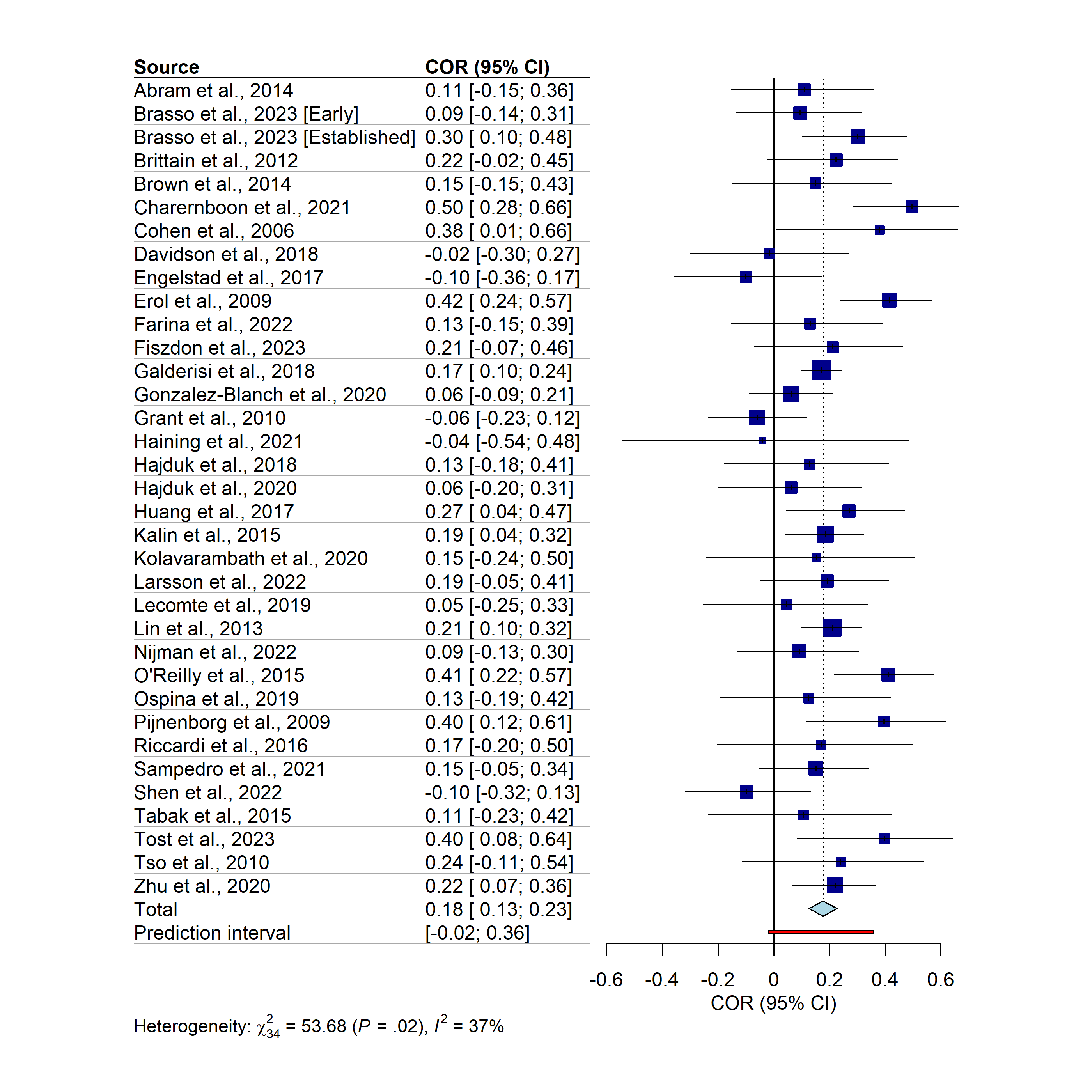


**Fig. S1.** Emotion Processing and Social Functioning – Forest Plot


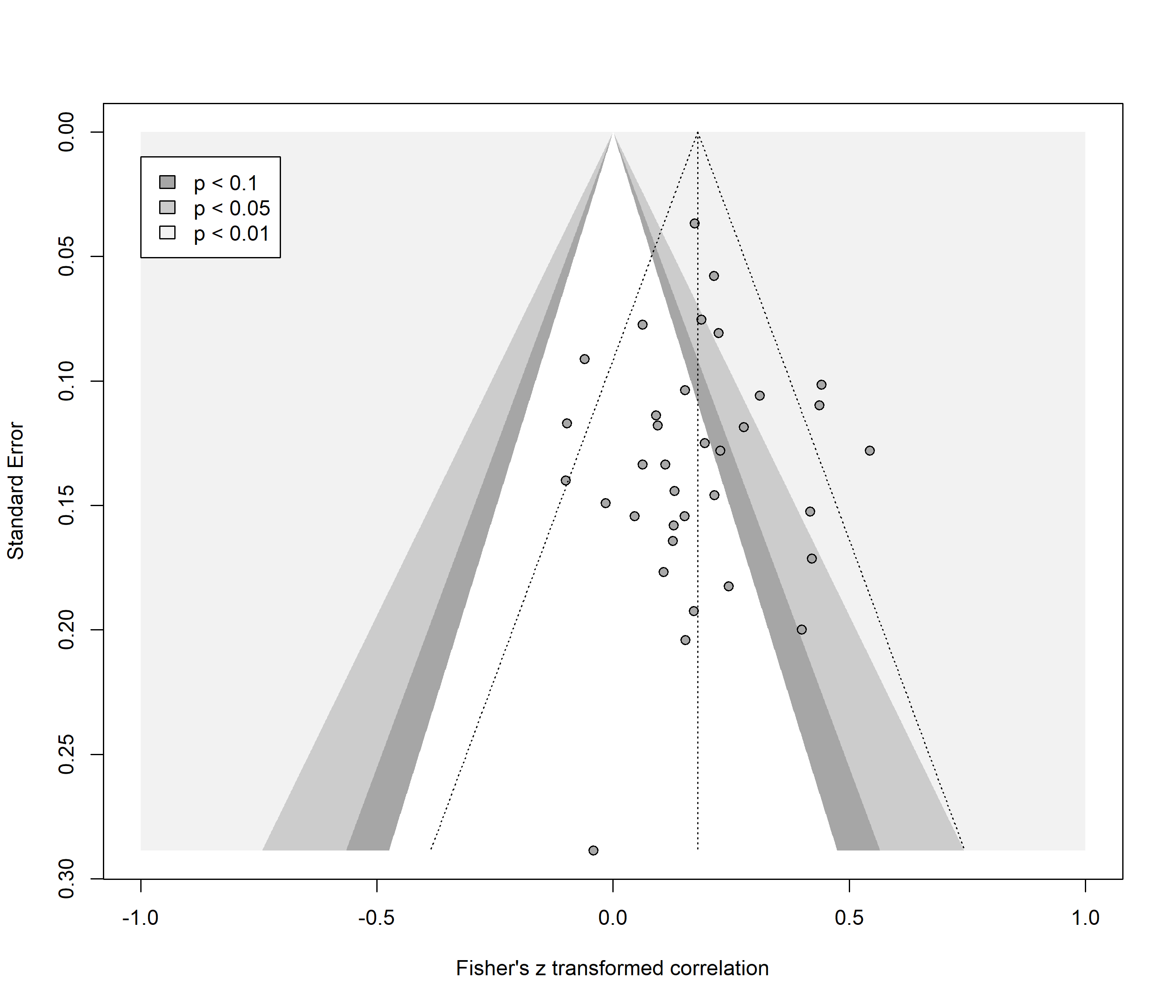


**Fig. S2.** Emotion Processing and Social Functioning – Funnel Plot


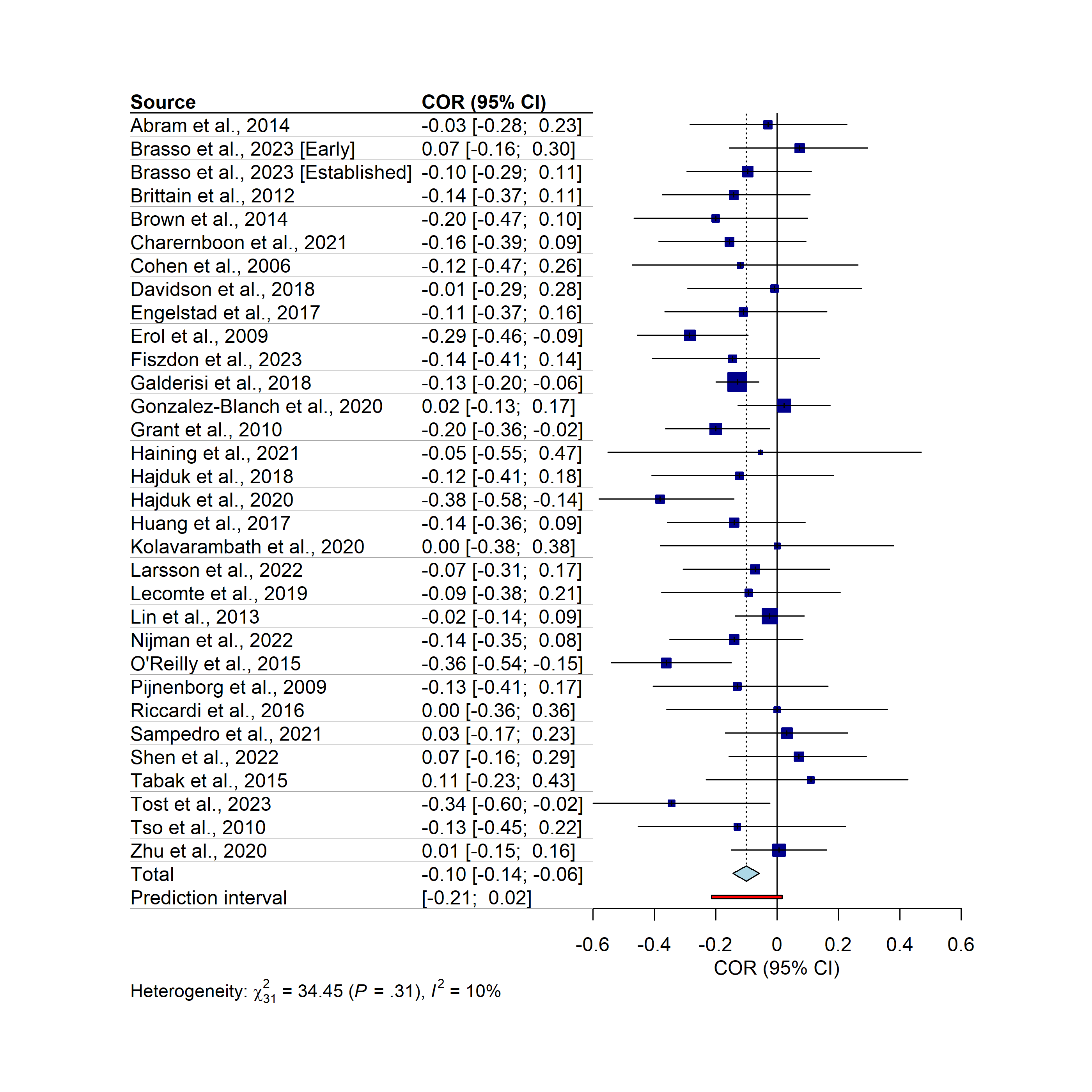


**Fig. S3.** Emotion processing and Positive Symptoms – Forest Plot


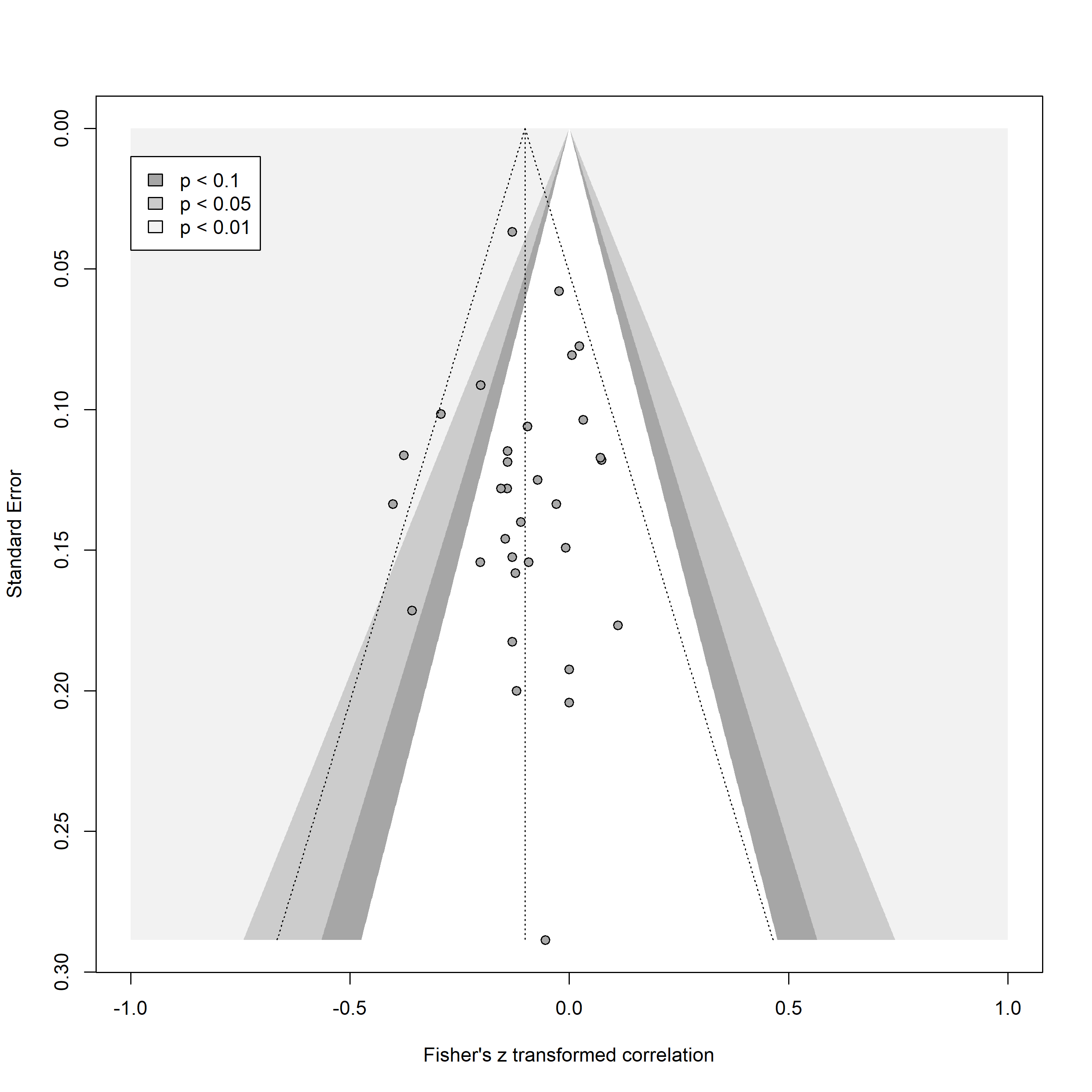


**Fig. S4.** Emotion Processing and Positive Symptoms – Funnel Plot


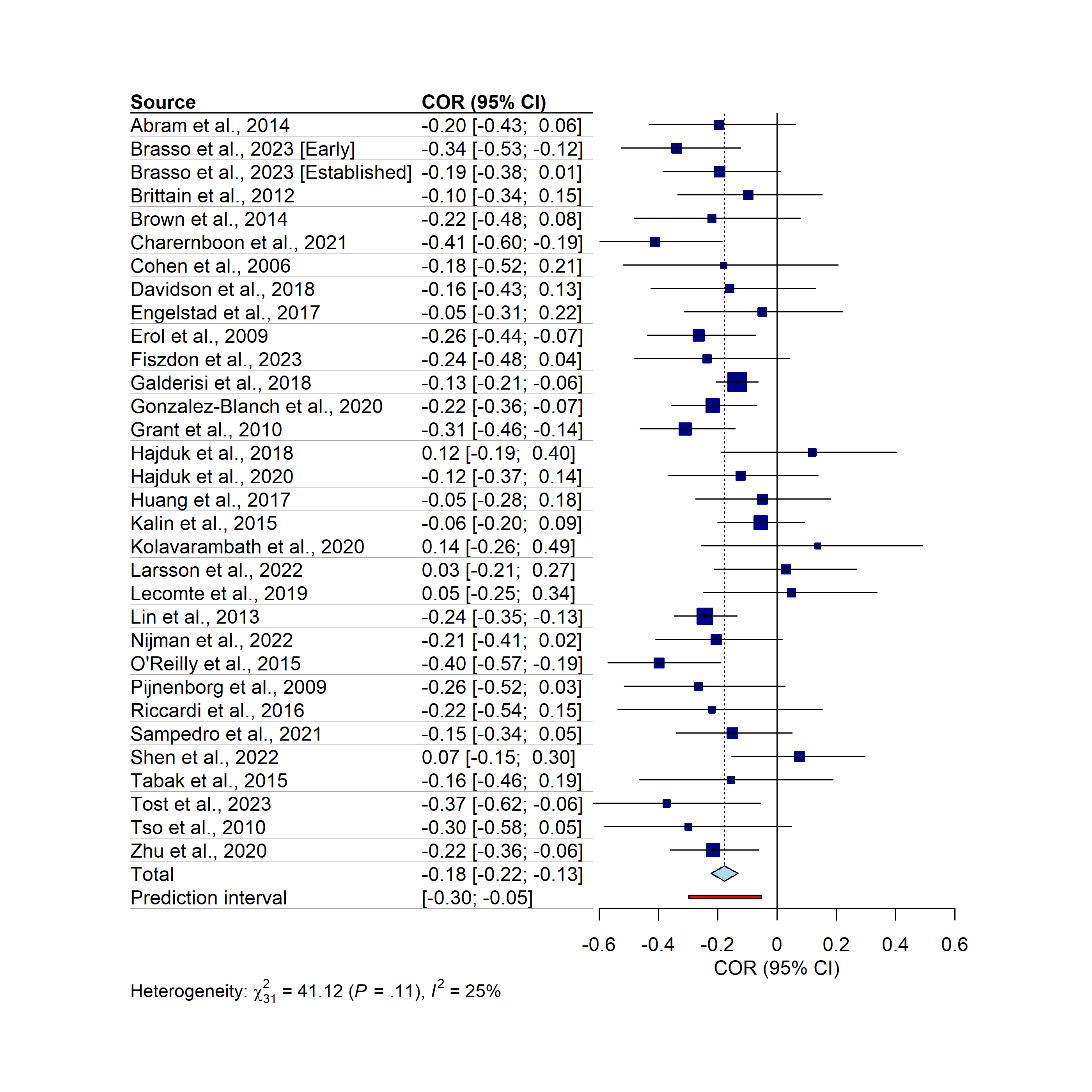


**Fig. S5.** Emotion Processing and Negative Symptoms – Forest Plot


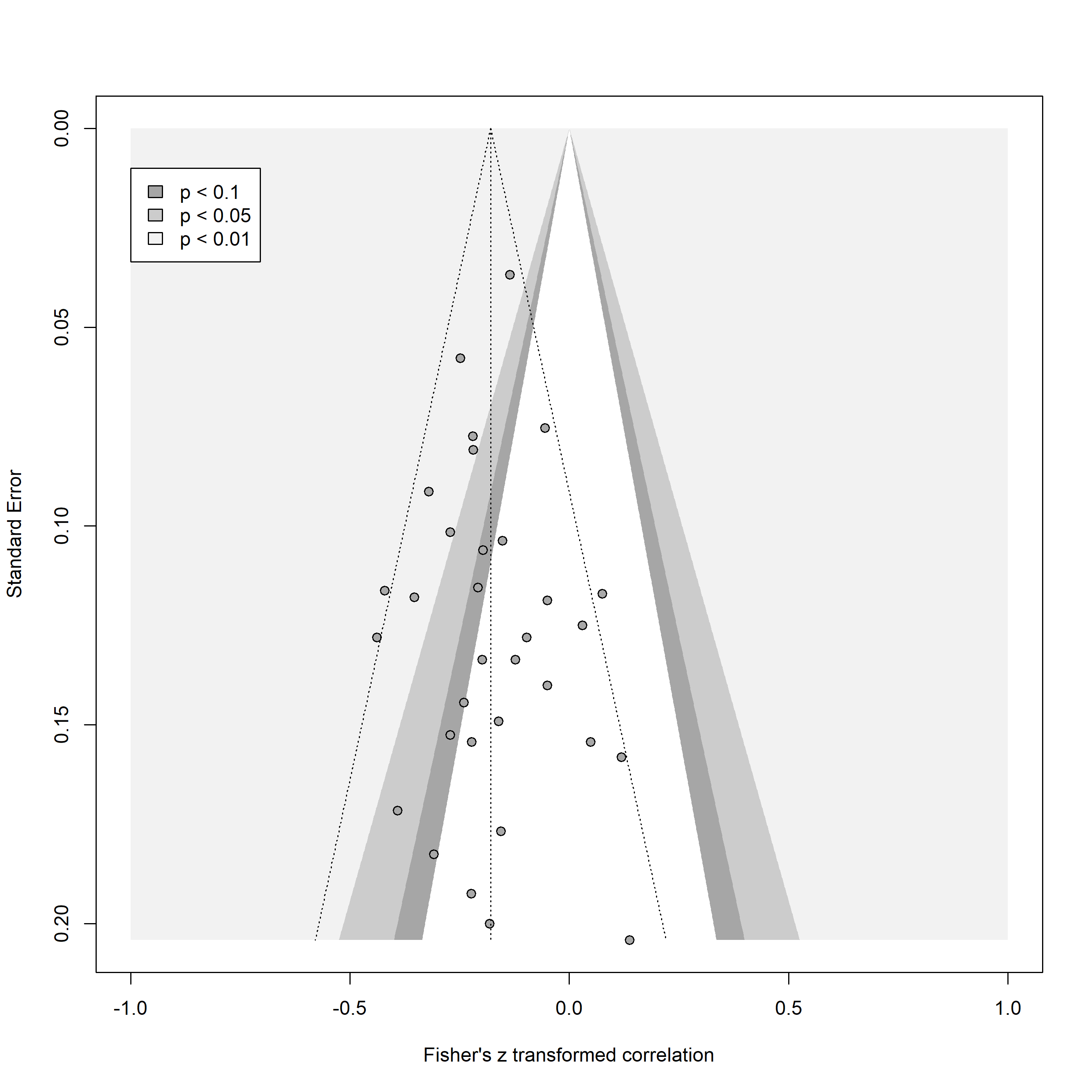


**Fig. S6.** Emotion Processing and Negative Symptoms – Funnel Plot


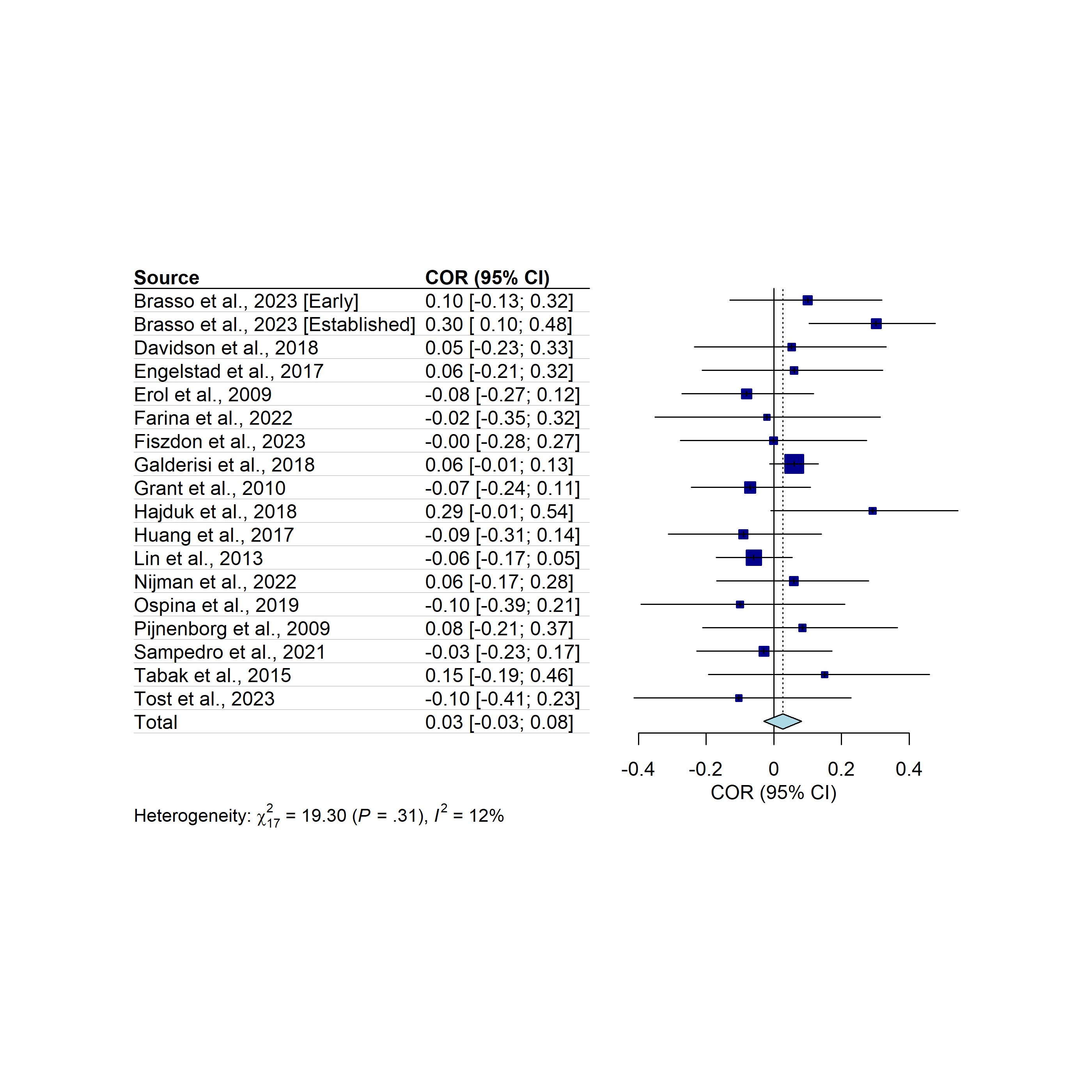


**Fig. S7.** Emotion Processing and Depressive Symptoms – Forest Plot


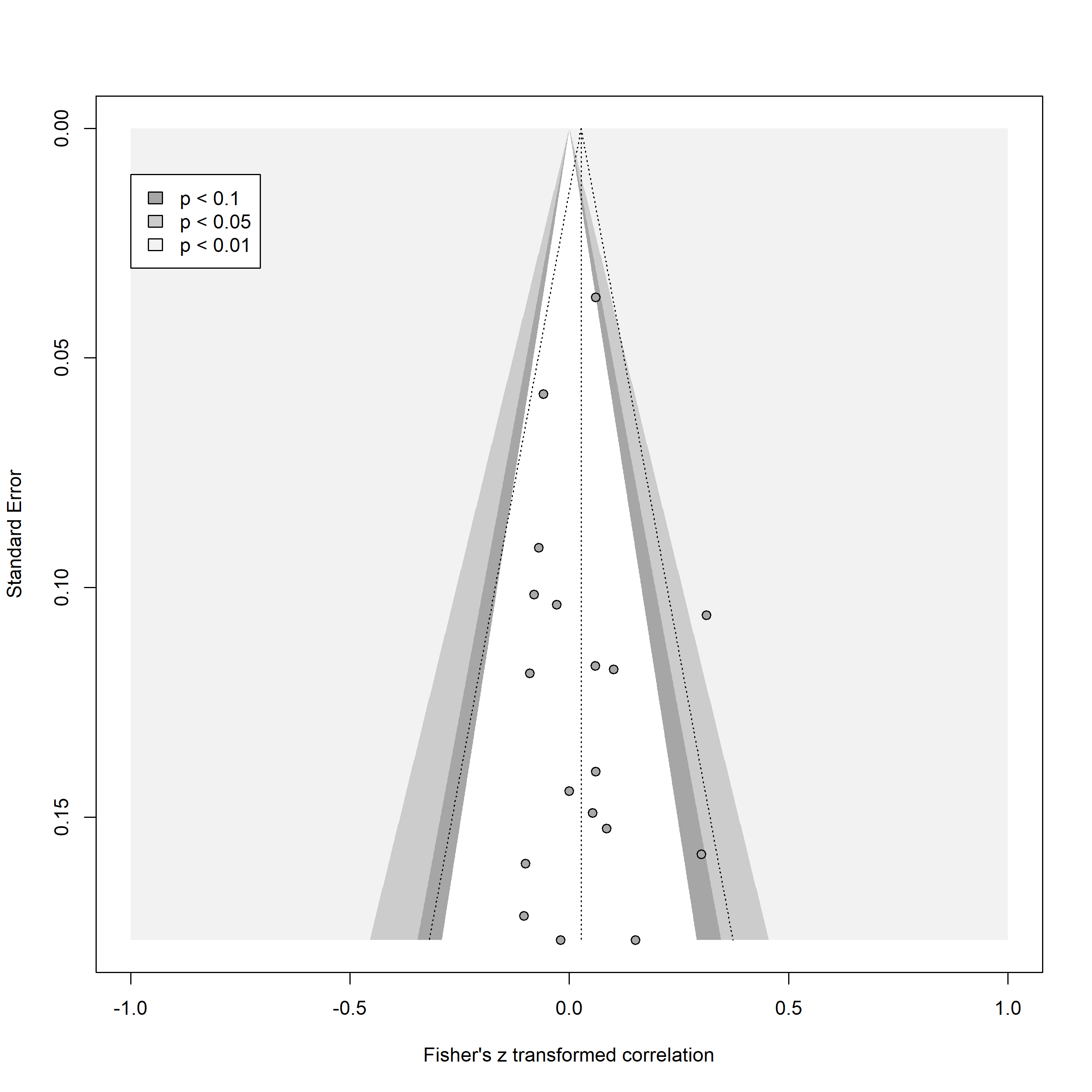


**Fig. S8.** Emotion Processing and Depressive Symptoms – Funnel Plot


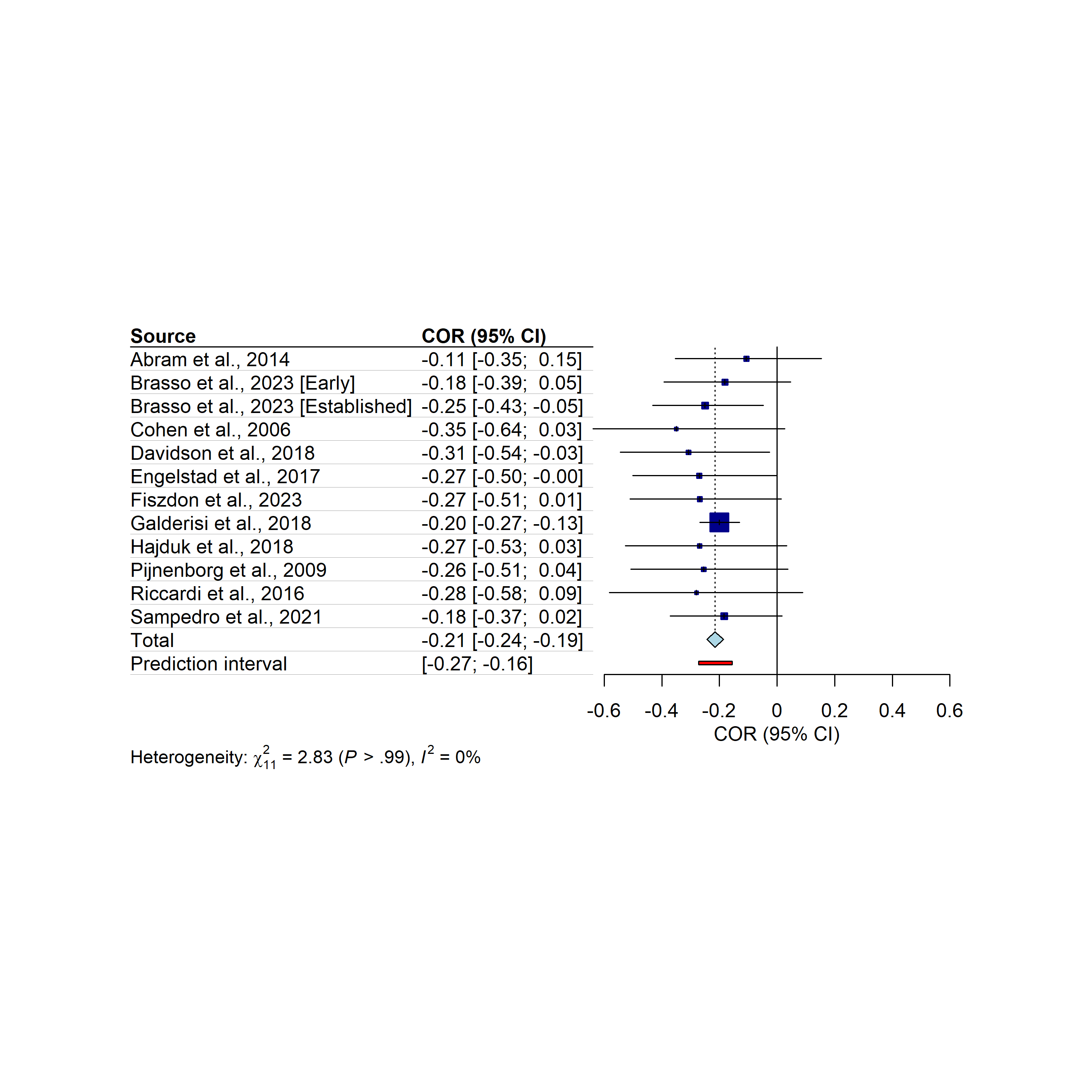


**Fig. S9.** Emotion Processing and Disorganisation Symptoms – Forest Plot


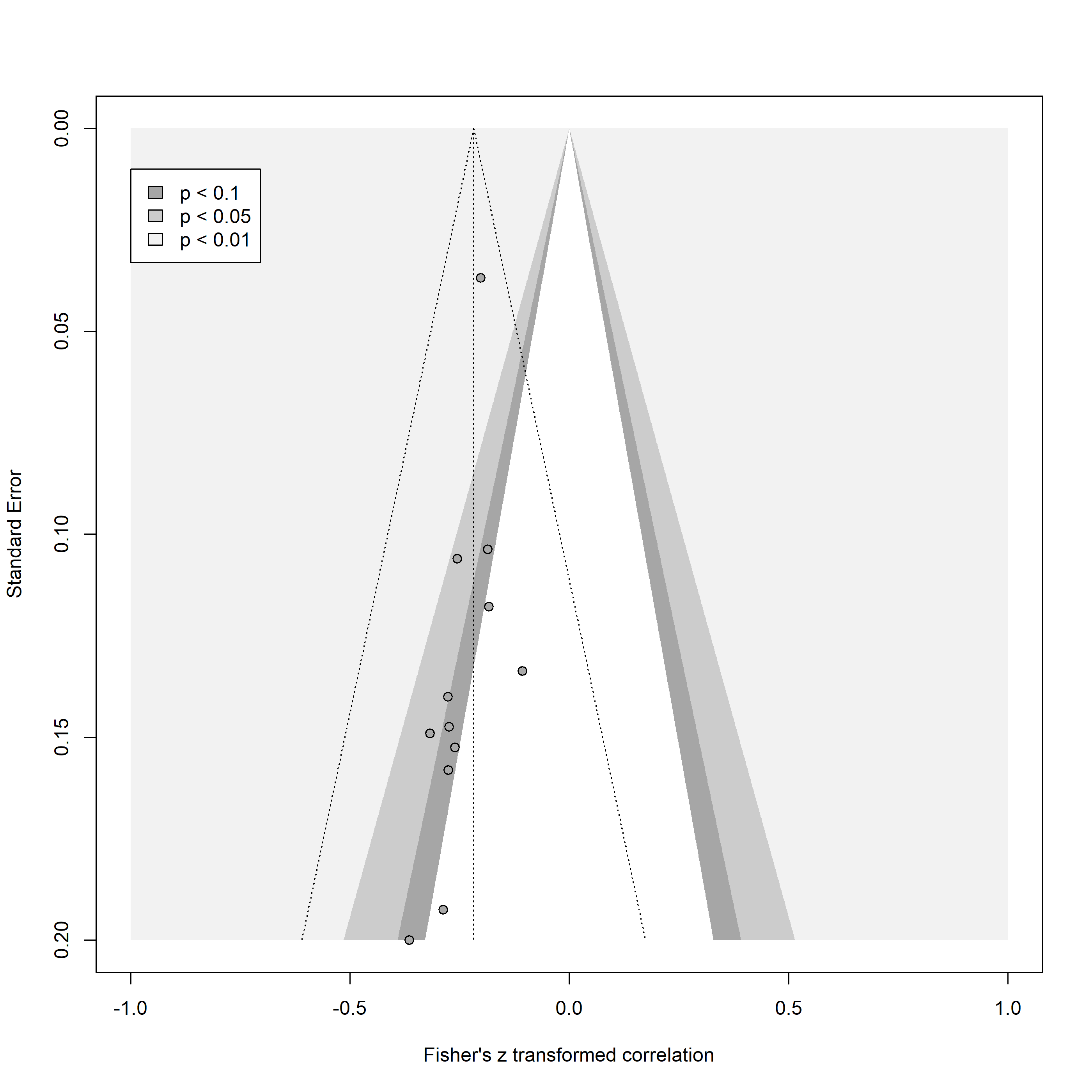


**Fig. S10.** Emotion Processing and Disorganisation Symptoms – Funnel Plot


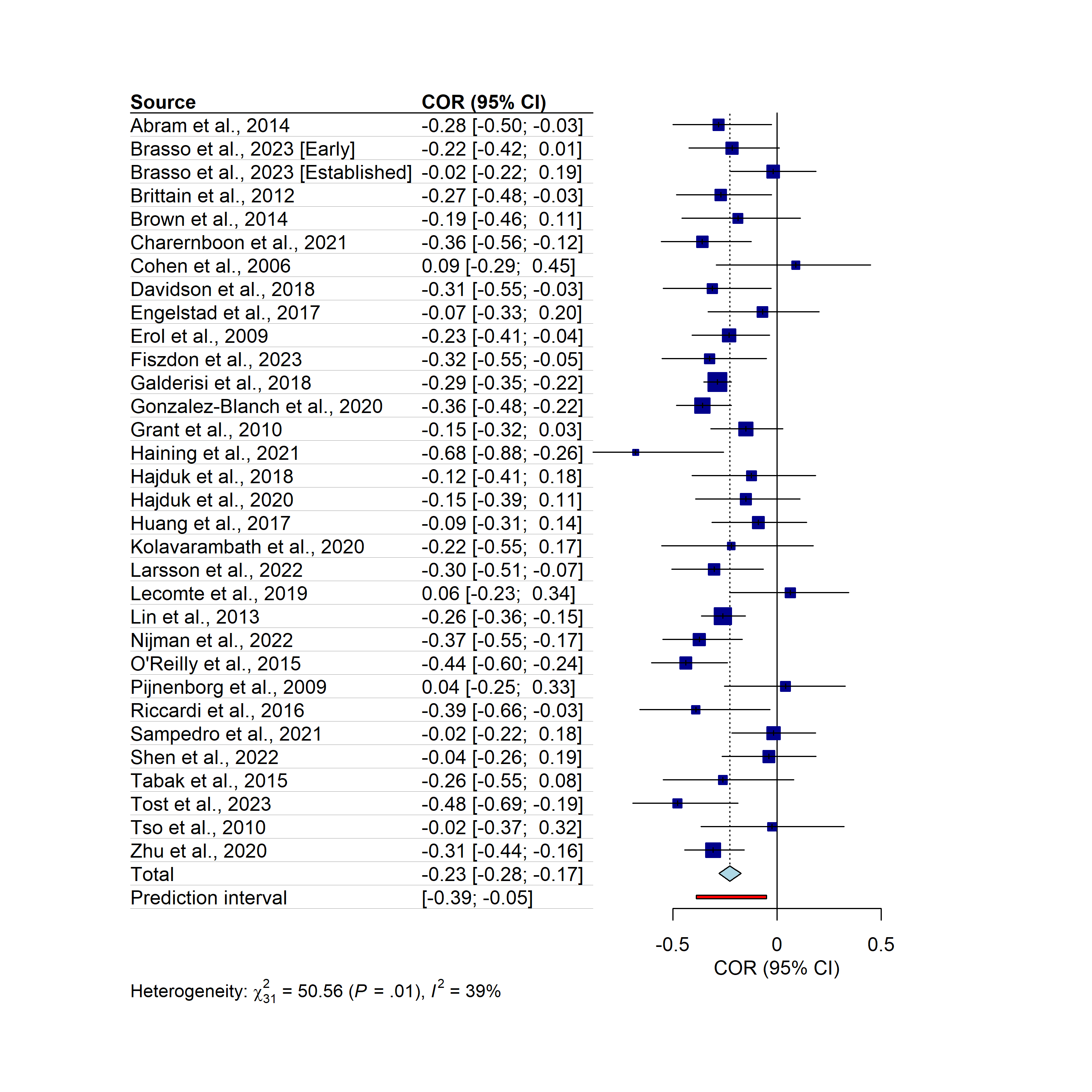


**Fig. S11.** Positive Symptoms and Social Functioning– Forest Plot


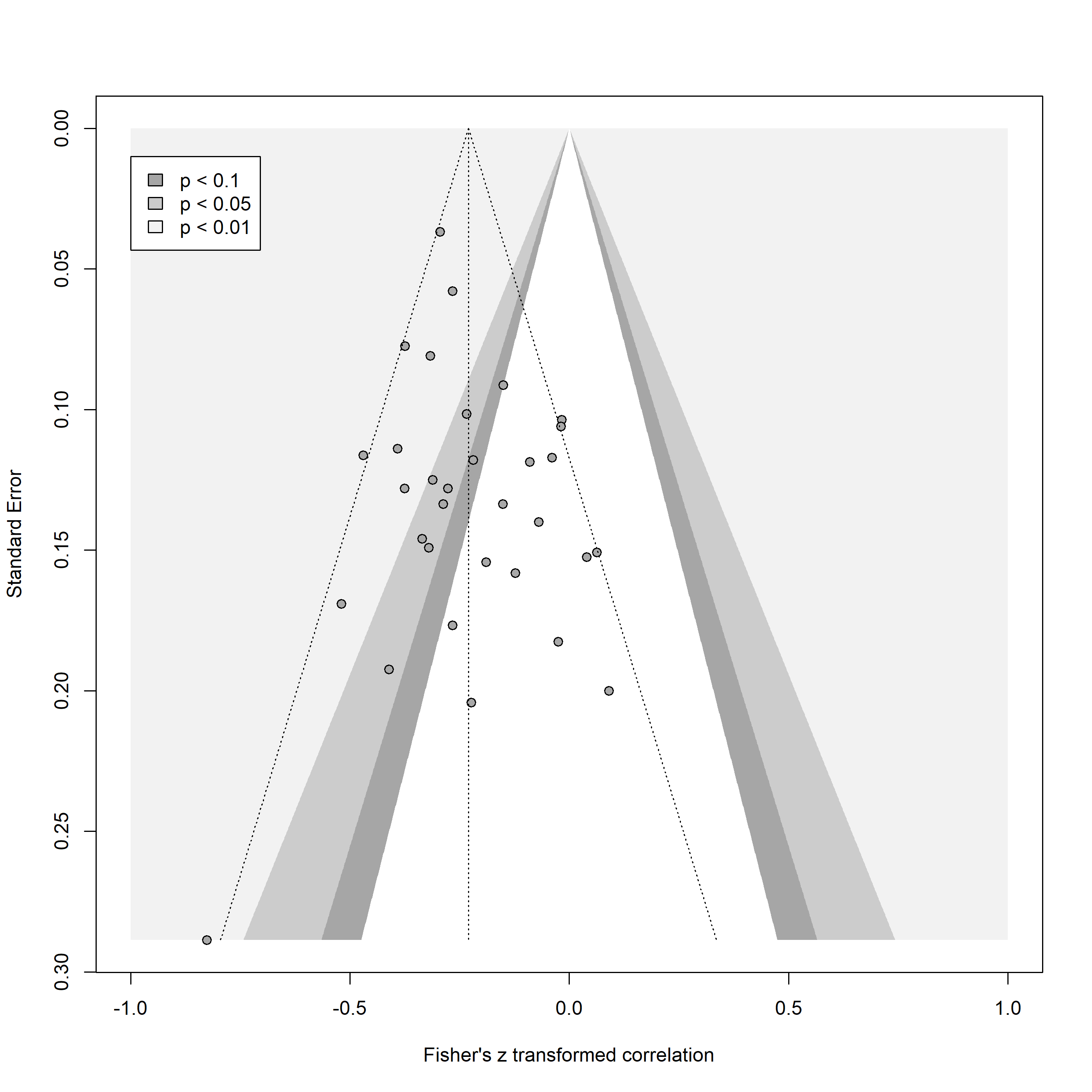


**Fig. S12.** Positive Symptoms and Social Functioning– Funnel Plot


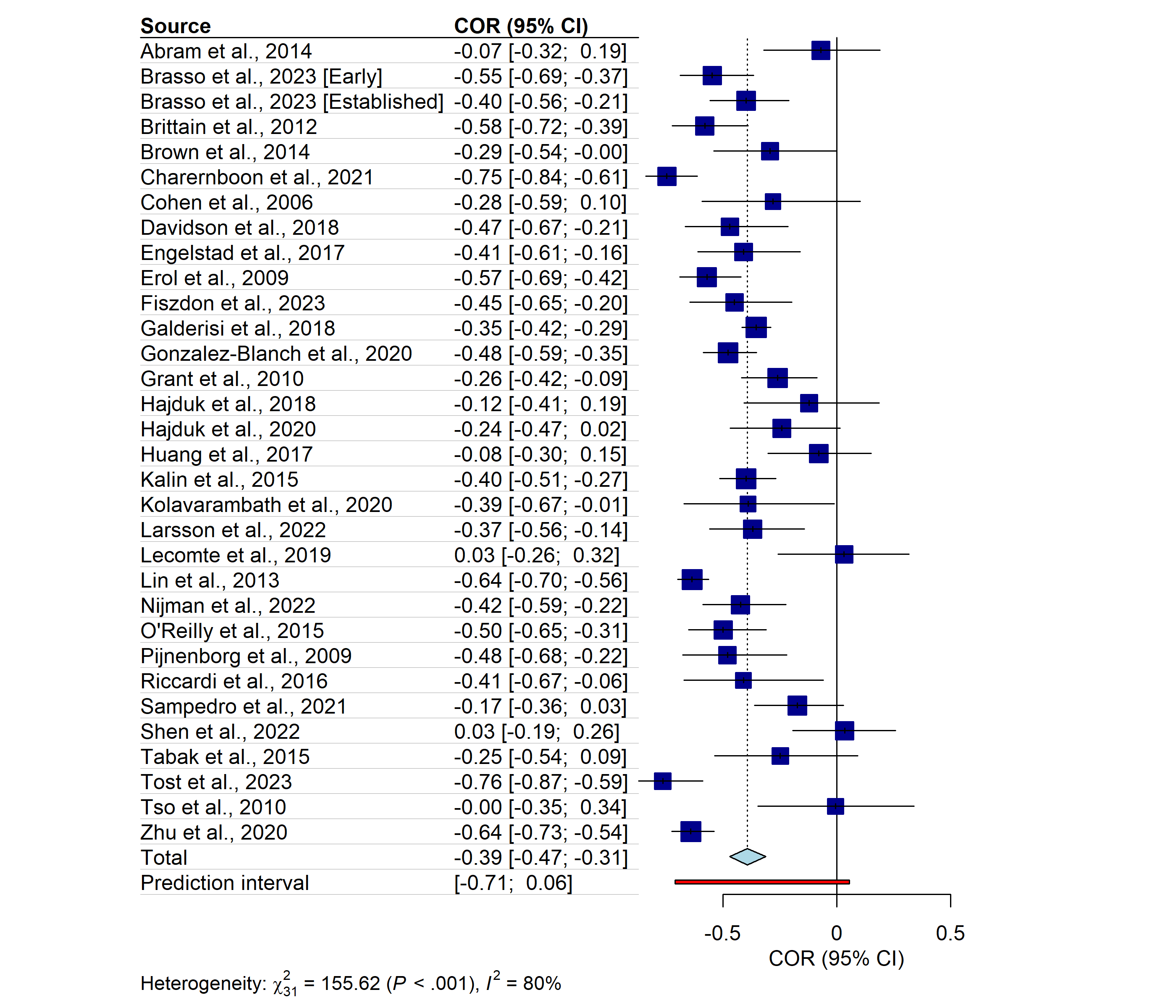


**Fig. S13.** Negative Symptoms and Social Functioning– Forest Plot


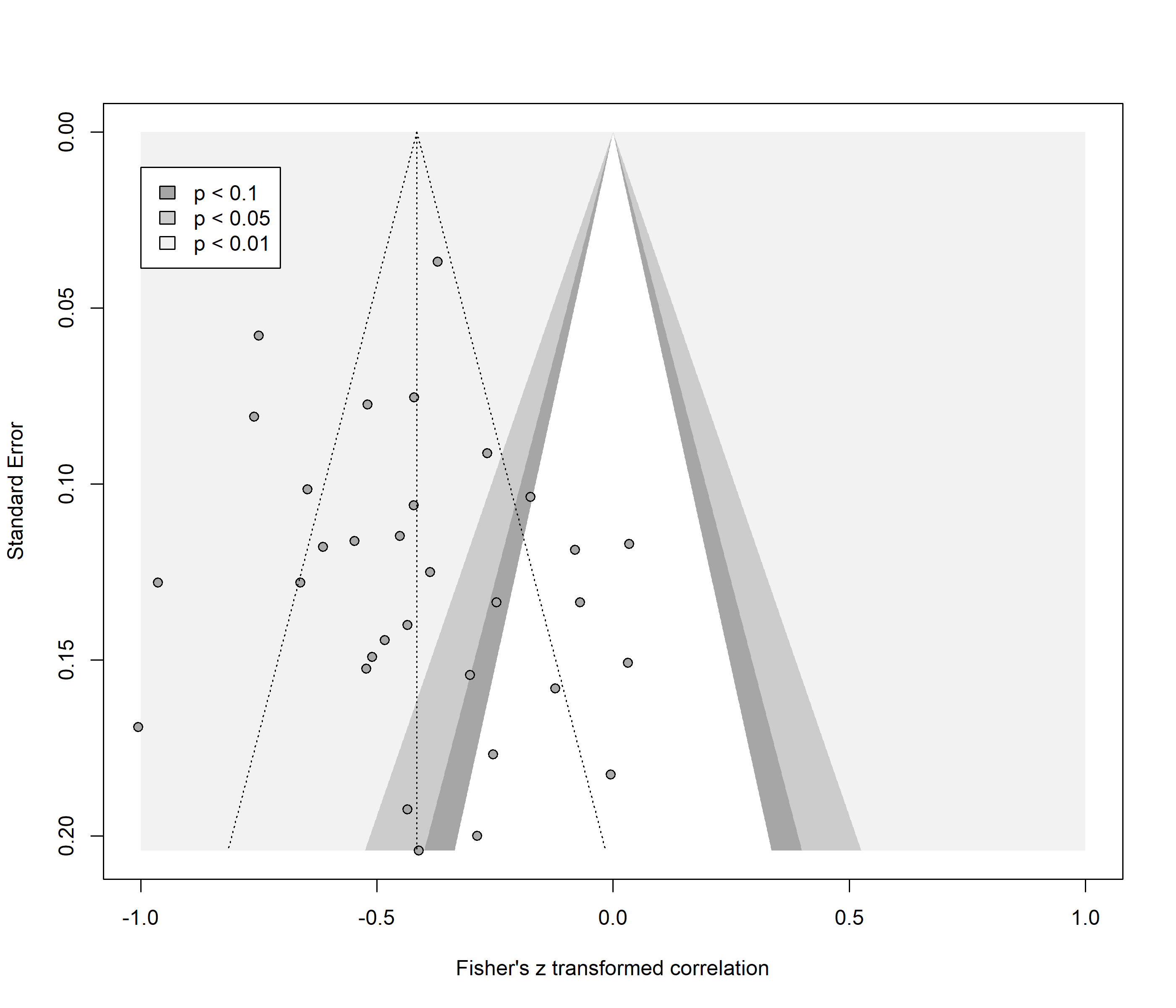


**Fig. S14.** Negative Symptoms and Social Functioning– Funnel Plot


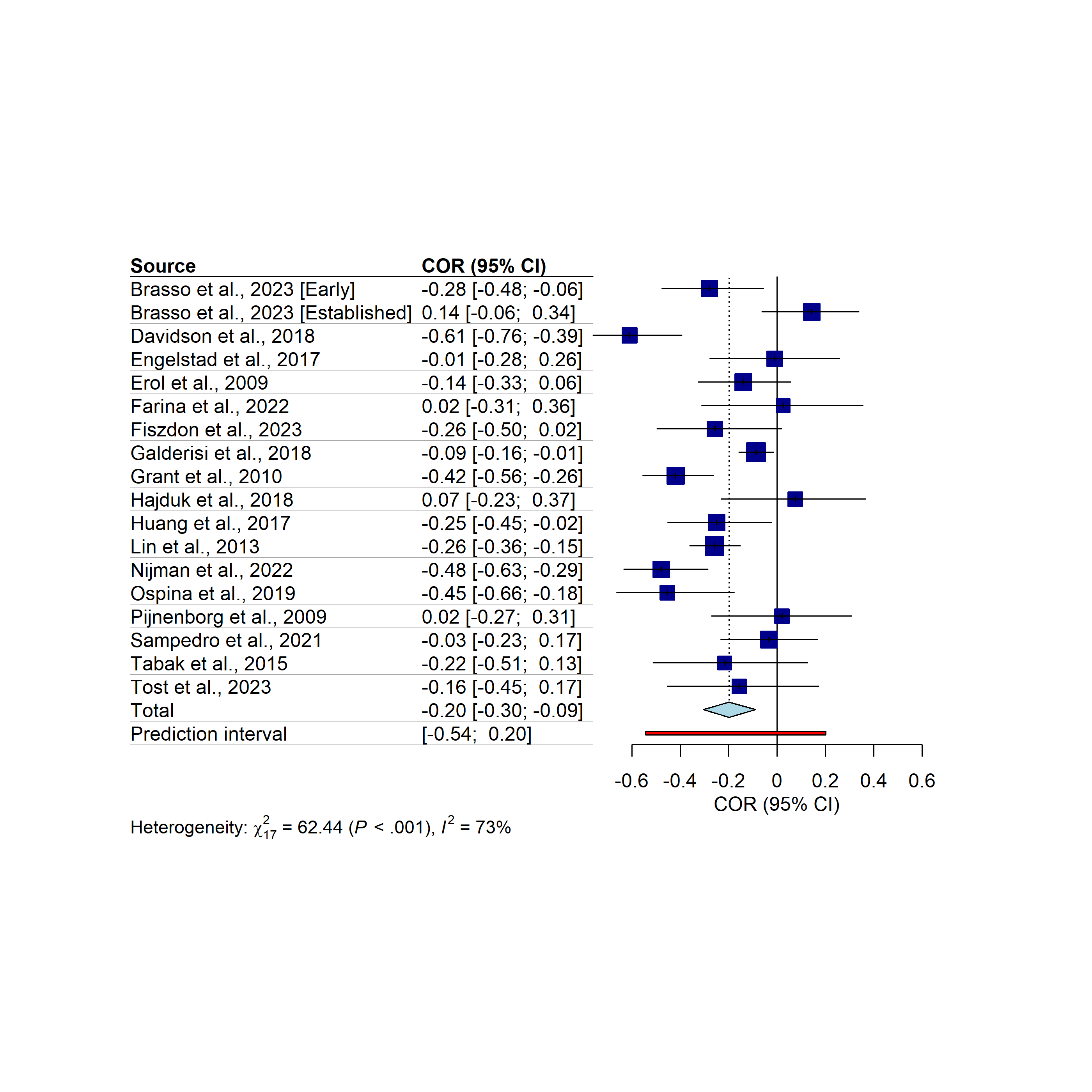


**Fig. S15.** Depressive Symptoms and Social Functioning– Forest Plot


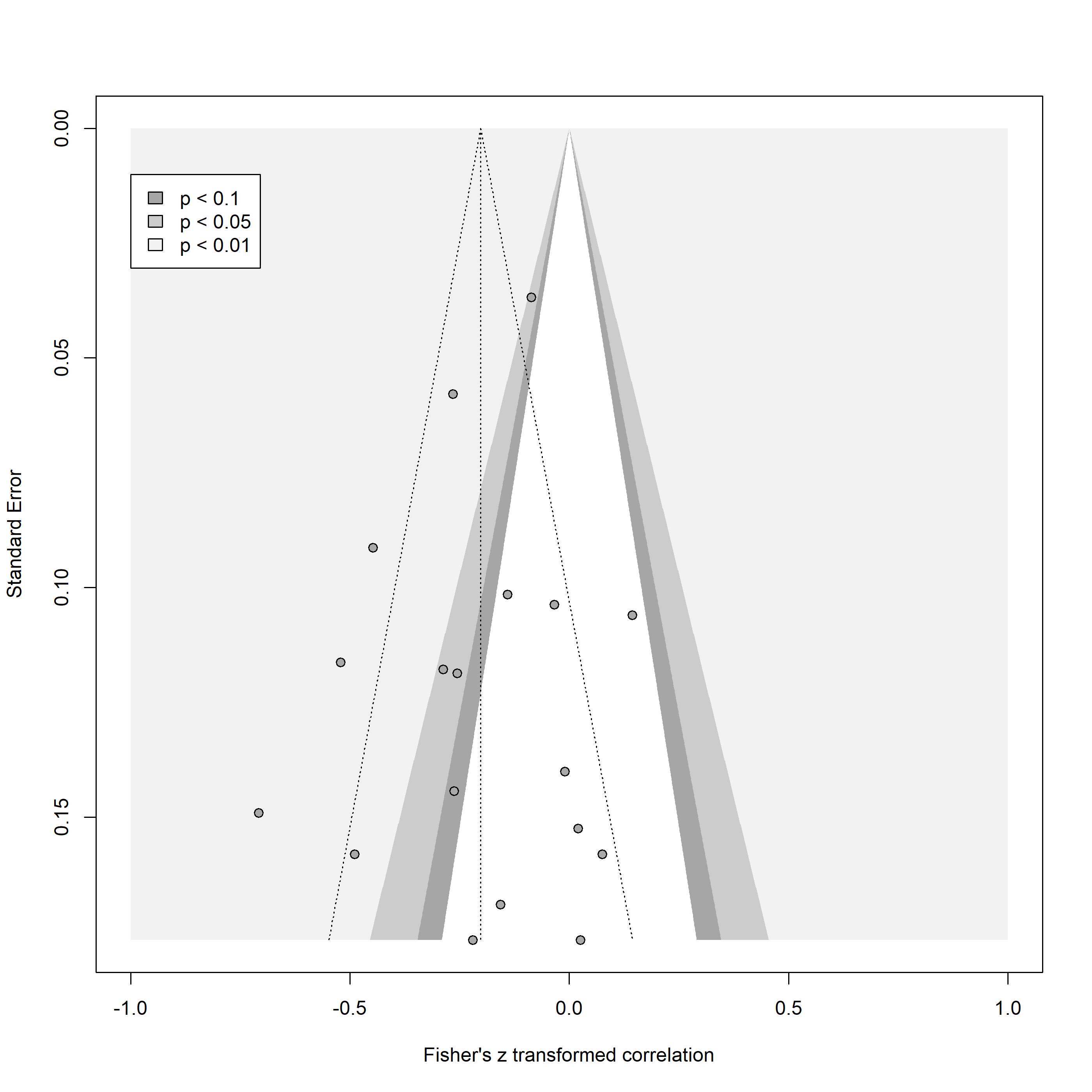


**Fig. S16.** Depressive Symptoms and Social Functioning– Funnel Plot


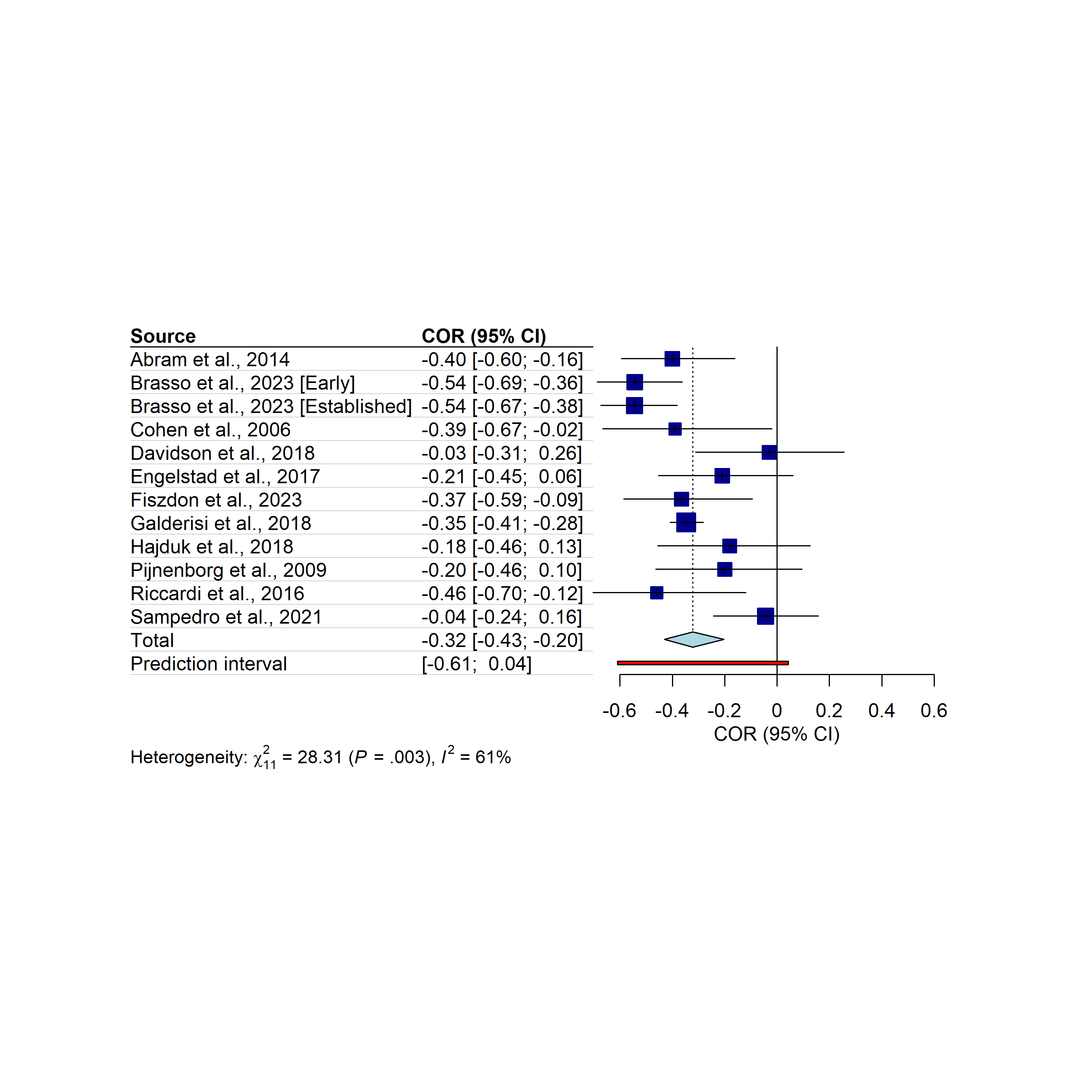


**Fig. S17.** Disorganization Symptoms and Social Functioning– Forest Plot


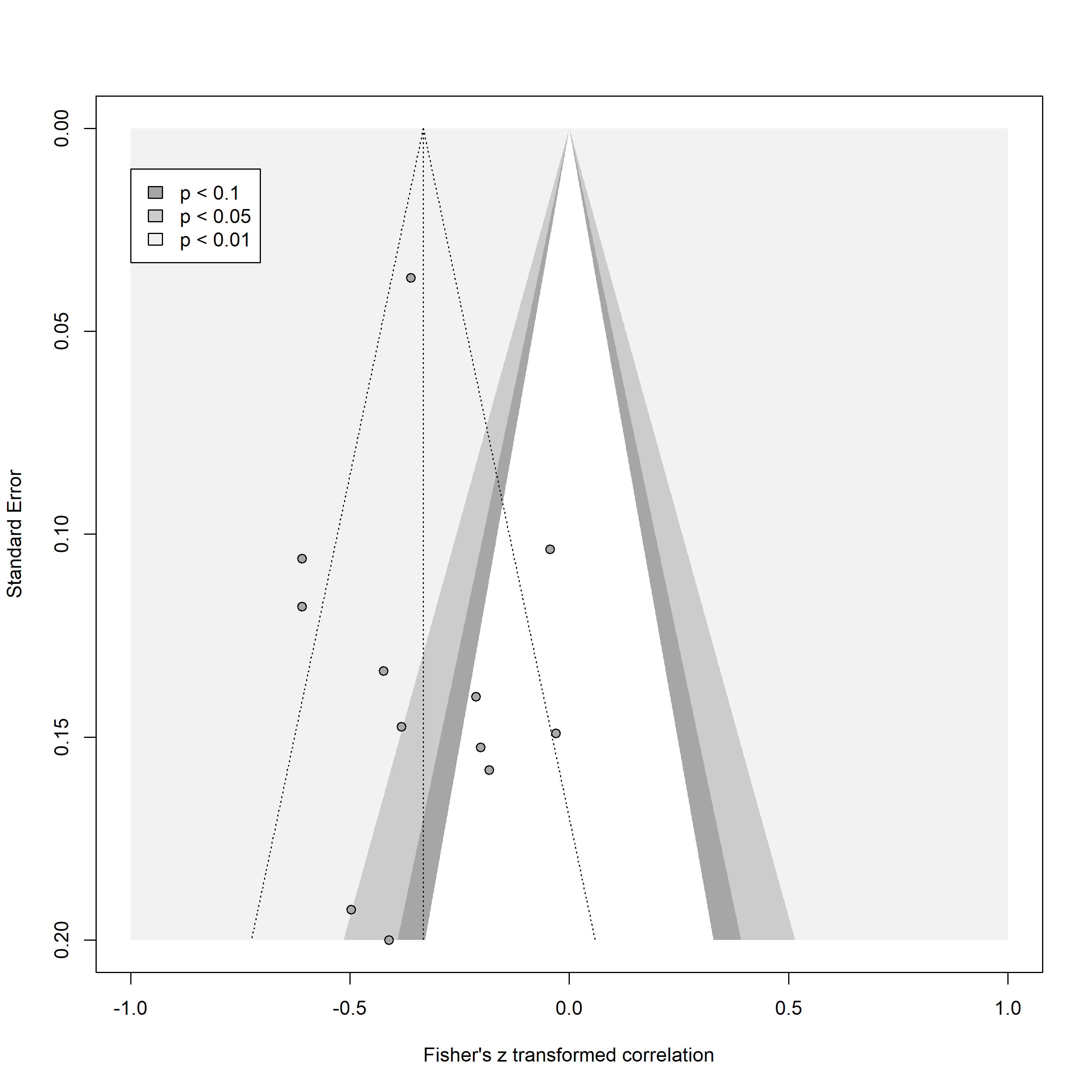


**Fig. S18.** Disorganization Symptoms and Social Functioning– Funnel Plot

**Table S11**. Moderator Analysis (Meta-Regression) for the Nine Outcome Correlation Pairs

| **Correlation /** |  |  | **95% CI** | |  |  |
| --- | --- | --- | --- | --- | --- | --- |
| **Moderator Variable** | **k** | **β** | **lower** | **upper** | ***p*** | **R^2^ (%)** |
| **EP – Social functioning** |  |  |  |  |  |  |
| Age | 35 | 0.00 | 0.00 | 0.01 | .413 | 1.85% |
| Male | 35 | 0.00 | 0.00 | 0.01 | .676 | 0.00% |
| Setting | 35 | 0.01 | -0.11 | 0.14 | .808 | 0.00% |
| Diagnosis | 32 | 0.11 | 0.00 | 0.22 | .056 | 19.16% |
| AP dose | 11 | 0.00 | 0.00 | 0.00 | .348 | 0.00% |
| Methodological Quality | 35 | 0.04 | -0.02 | 0.10 | .142 | 14.97% |
| **EP – Positive symptoms** |  |  |  |  |  |  |
| Age | 32 | 0.00 | -0.01 | 0.00 | .303 | 12.00% |
| Male | 32 | 0.00 | -0.01 | 0.00 | .572 | 0.00% |
| Setting | 29 | 0.01 | -0.08 | 0.10 | .777 | 0.00% |
| Diagnosis | 30 | 0.04 | -0.05 | 0.13 | .367 | 0.00% |
| AP dose | 10 | 0.00 | 0.00 | 0.00 | .181 | 3.25% |
| Methodological Quality | 32 | 0.02 | -0.03 | 0.07 | .414 | 0.00% |
| **EP – Negative symptoms** |  |  |  |  |  |  |
| Age | 32 | 0.00 | -0.01 | 0.01 | .807 | 0.00% |
| Male | 32 | 0.00 | -0.01 | 0.00 | .267 | 0.00% |
| Setting | 29 | 0.03 | -0.08 | 0.13 | .621 | 0.00% |
| Diagnosis | 30 | -0.02 | -0.12 | 0.09 | .770 | 0.00% |
| AP dose | 10 | 0.00 | 0.00 | 0.00 | .471 | 0.00% |
| Methodological Quality | 32 | 0.01 | -0.05 | 0.06 | .791 | 0.00% |
| **EP – Depressive symptoms** |  |  |  |  |  |  |
| Age | 18 | 0.00 | -0.01 | 0.01 | .565 | 0.00% |
| Male | 18 | 0.00 | 0.00 | 0.01 | .490 | 0.00% |
| Setting | 17 | -0.08 | -0.19 | 0.03 | .140 | 65.98% |
| Diagnosis | 17 | 0.00 | -0.13 | 0.14 | .979 | 0.00% |
| AP dose^a^ | - | - | - | - | - | - |
| Methodological Quality | 18 | 0.01 | -0.05 | 0.08 | .618 | 0.00% |
| **EP – Disorganization symptoms** | |  |  |  |  |  |
| Age | 12 | 0.00 | -0.01 | 0.00 | .645 | 0.00% |
| Male | 12 | 0.00 | 0.00 | 0.01 | .423 | 0.00% |
| Setting | 12 | -0.03 | -0.12 | 0.06 | .441 | 0.00% |
| Diagnosis | 12 | 0.08 | 0.00 | 0.16 | .044 | 0.00% |
| AP dose^a^ | - | - | - | - | - | - |
| Methodological Quality | 12 | 0.02 | -0.02 | 0.06 | .314 | 0.00% |
| **Social functioning – Positive symptoms** | |  |  |  |  |  |
| Age | 32 | 0.00 | -0.01 | 0.01 | .981 | 0.00% |
| Male | 32 | 0.00 | 0.00 | 0.01 | .611 | 0.00% |
| Setting | 29 | 0.08 | -0.05 | 0.20 | .206 | 0.91% |
| Diagnosis | 30 | 0.10 | -0.02 | 0.23 | .094 | 7.90% |
| AP dose | 10 | 0.00 | 0.00 | 0.00 | .746 | 0.00% |
| Methodological Quality | 32 | -0.04 | -0.10 | 0.02 | .215 | 12.43% |
| **Social functioning – Negative symptoms** | |  |  |  |  |  |
| Age | 32 | 0.00 | -0.02 | 0.01 | .885 | 0.00% |
| Male | 32 | 0.00 | -0.01 | 0.01 | .714 | 0.00% |
| Setting | 29 | 0.06 | -0.15 | 0.26 | .576 | 0.00% |
| Diagnosis | 30 | -0.02 | -0.23 | 0.18 | .825 | 0.00% |
| AP dose | 10 | 0.00 | 0.00 | 0.00 | .707 | 0.00% |
| Methodological Quality | 32 | -0.04 | -0.15 | 0.06 | .420 | 0.00% |
| **Social functioning – Depressive symptoms** | | |  |  |  |  |
| Age | 18 | -0.01 | -0.03 | 0.00 | .132 | 5.24% |
| Male | 18 | 0.01 | -0.01 | 0.02 | .319 | 0.00% |
| Setting | 17 | 0.03 | -0.23 | 0.28 | .830 | 0.00% |
| Diagnosis | 17 | 0.22 | 0.01 | 0.44 | .044 | 33.81% |
| AP dose^a^ | - | - | - | - | - | - |
| Methodological Quality | 18 | 0.00 | -0.13 | 0.12 | .987 | 0.00% |
| **Social functioning – Disorganization symptoms** | | |  |  |  |  |
| Age | 12 | 0.01 | -0.01 | 0.02 | .466 | 0.00% |
| Male | 12 | 0.00 | -0.01 | 0.01 | .912 | 0.00% |
| Setting | 12 | 0.22 | -0.04 | 0.47 | .087 | 42.01% |
| Diagnosis | 12 | -0.19 | -0.45 | 0.08 | .148 | 13.06% |
| AP dose^a^ | - | - | - | - | - | - |
| Methodological Quality | 12 | -0.11 | -0.23 | 0.00 | .052 | 55.71% |
| ^a^ Meta-regression not conducted, as *k* < 10.  *Note:* EP = emotion processing; Setting = outpatient v inpatient; Diagnosis = 100% schizophrenia sample v broader psychosis sample | | | | | | |


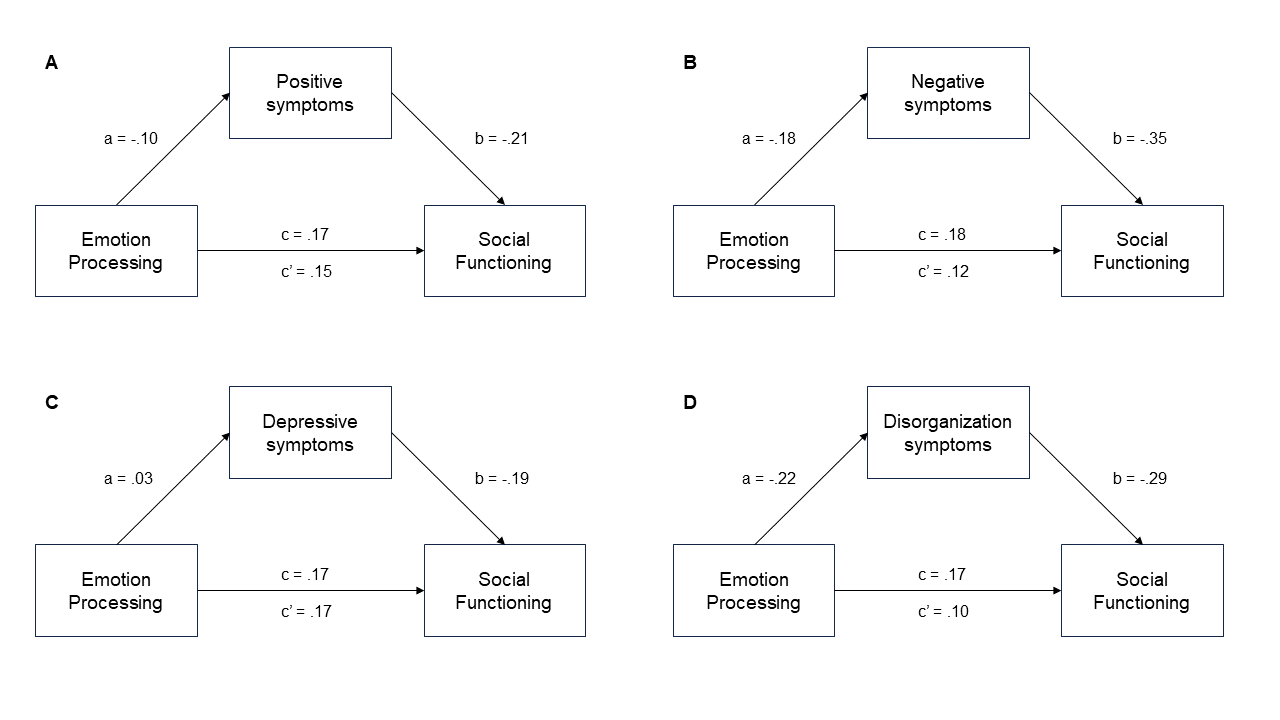


**Fig. S19.** Mediation analyses examining mediation of the relationship between emotion processing and social functioning by the four symptom domains: (a) positive symptoms, (b) negative symptoms, (c) depressive symptoms, (d) disorganization symptoms. *Note*: c = total effect, c’ = direct effect (i.e., the effect controlled for the mediator), and the product of a*b = indirect effect.

**Table S12.** Exploratory Meta-analyses for the Two Subdomains of Negative Symptoms

| **Correlation /** |  |  |  |  | **Heterogeneity** | | |
| --- | --- | --- | --- | --- | --- | --- | --- |
| **Negative symptom domain** | ***k*** | ***r*** | ***95% CI*** | ***p*** | ***I^2^ (%)*** | ***Q*** | ***p*** |
| **EP – Negative symptoms** | | | | |  |  |  |
| Expressive symptoms | 4 | -.21 | [-.34, -.08] | .015 | 9.2 | 3.30 | .347 |
| Experiential symptoms | 4 | -.16 | [-.36, .06] | .108 | 46.3 | 5.59 | .134 |
| **Negative symptoms – Social functioning** | | | | |  |  |  |
| Expressive symptoms | 4 | -.39 | [-.56, -.18] | .011 | 49.4 | 5.93 | .115 |
| Experiential symptoms | 4 | -.40 | [-.53, -.25] | .004 | 26.5 | 4.08 | .253 |
| *Note:* EP, emotion processing | | | | | | | |

# **Sensitivity Analyses**

Sensitivity analyses were conducted to assess the robustness of the study findings to decisions made regarding classification of outcomes and stage of illness, and to the effects of potential outliers. The results of the sensitivity analyses for the meta-analyses on the associations between outcome pairs can be found in Table S13.

First, the effects of potential outliers were examined for the nine correlation pairs. Following recommendations by Harrer et al.^50^, outliers and influential cases were only examined for meta-analyses where *I^2^* > 50%, using a combination of these methods: (i) finding confidence intervals of individual studies which did not overlap with the confidence interval of the pooled effect; (ii) finding standardized residuals > 1.96, and (iii) examining Baujat plots^51^, which plot Cochran’s Q of each study against the studies influence on the **pooled effect size**. Potential outliers were identified for the social functioning-negative symptoms and social functioning-depressive symptoms correlations in the established psychosis cohorts. When potential outliers were removed for the social functioning and negative symptoms correlation, the estimated correlation was very similar (*r* = -.38 compared to *r* = -.39 with potential outliers included), and while the between-study heterogeneity was reduced somewhat, moderate between-study heterogeneity remained (*I^2^ =* 44.80%). Removal of potential outliers for the social functioning and depressive symptoms correlation did not change the estimated effect size (*r* = -.20)., with only a small reduction in heterogeneity (< 10% difference in *I^2^*). Given the limited impact of potential outliers on results, outliers were not removed for the primary meta-analyses of the nine correlation pairs or for the further analyses.

Next, we examined the effects of the classification approach we took for the three outcomes of interest. There was a wide array of EP measures used throughout the included studies. We examined the effects of including only those with well-assessed psychometric properties recommended for use in the SCOPE project (i.e. Penn Emotion recognition task and BLERT) and the Mayer-Salovey-Caruso Emotional Intelligence Test (MSCEIT), which was not examined in the SCOPE project due to its already well-validated psychometric properties. The estimated effects sizes were stable when using SCOPE measures and MSCEIT: notably, the estimated correlation between EP and social functioning remained the same (*r* = .18), and while there were some small differences regarding the EP-symptom correlations, the largest difference was a reduction in the strength of the EP-disorganization association (*r* = -.16 compared to *r* = -.21 in primary analysis). This observed difference may reflect some instability due to the small number of studies reporting the Ep-disorganization correlation.

In the primary analysis, we took an inclusive approach to the classification of the positive symptom domain. Measurement of positive symptoms is complicated by the inclusion of disorganization items in some scales (e.g., ‘conceptual disorganization’ in the Positive and Negative Syndrome Scale [PANSS] positive subscale). To explore disorganization as a potential confound, we followed an approach used by Ventura et al.^52^ to divide positive symptoms into those that solely measured reality distortion symptoms (i.e., hallucinations and delusions) and those that combined reality distortion and disorganization symptoms (termed total positive symptoms). Their correlations with EP and social functioning were then examined separately. The estimated association with EP was similar for total positive symptoms (*r* = -.12) and reality distortion (*r* = -.10), though the between-study heterogeneity was smaller when including reality distortion (*I^2^* = 0%) compared to total positive symptom measures (*I^2^* = 41%). The negative association with social functioning was estimated to be stronger for total positive symptoms (*r* = -.26) than reality distortion (*r* = -.17).

Depressive symptoms were measured using either dedicated depression measures (e.g., Calgary Depression Scale for Schizophrenia [CDSS]^53^) or factor-derived scores from psychotic symptom scales (e.g., the ‘depression/anxiety’ factor derived from Wallwork’s 5-factor solution of PANSS^54^). Again, we used an inclusive approach in the primary analysis and examined correlations with EP and social functioning for depressive symptom scale scores and factor-derived depressive symptom scores separately in sensitivity analysis. Results were very similar when using all three approaches to classification of depressive symptoms (i.e., including both depression scale scores and factor-derived scores, depression scale scores only, or factor-derived scores only).

We also assessed the impact of the decision to include studies that used global functioning assessment of functioning (GAF) to assess social functioning, as this measure may have spuriously high correlations with symptoms. There were no noticeable differences in results when cohorts using GAF as a measure of social functioning (*k* = 5) were removed.

Finally, we conducted some additional sensitivity analyses for our mediation analyses. These were guided by the findings of the previously reported analyses. Given the confounding effects of disorganization items on positive symptom associations demonstrated in the earlier sensitivity analyses, we tested mediation of the relationship between EP and social functioning by positive symptoms when including reality distortion measures only, in the established psychosis group. When excluding positive symptom measures that encompassed disorganization items, the mediating effect of positive symptoms (i.e., reality distortion) on the relationship between EP and social functioning in the established psychosis group was smaller but remained significant (β_indirect_ = .01, 95% CI: [.005, .03]). Given the significant differences in the size of the relationship between EP and negative symptoms for the different EP task levels, we tested mediation by negative symptoms for lower- and higher-level tasks separately. Negative symptoms mediated the relationship between lower-level EP tasks and social functioning (β_indirect_ = .05, 95%CI: [.03, .07]) and between higher-level EP tasks and social functioning (β_indirect_ = .09, 95% CI: [.06, .13]).

**Table S13.** Sensitivity Analyses Results

| **Correlation /** |  |  |  |  | **Heterogeneity** | | |
| --- | --- | --- | --- | --- | --- | --- | --- |
| **Sensitivity Analysis** | ***k*** | ***r*** | **95% CI** | **p** | ***I^2^*** | ***Q*** | ***p*** |
| **EP - Social Functioning** |  |  |  |  |  |  |  |
| **Primary Analysis** | 35 | .18 | [.13, .23] | < .001 | 36.67% | 53.68 | .017 |
| SCOPE measures + MSCEIT only | 17 | .18 | [.13, .22] | < .001 | 9.70% | 17.71 | .341 |
| GAF removed | 30 | .18 | [.12, .23] | < .001 | 44.80% | 52.54 | .005 |
| **EP - Positive symptoms** |  |  |  |  |  |  |  |
| **Primary Analysis** | 32 | -.10 | [-.14, -.06] | < .001 | 10.02% | 34.45 | .306 |
| SCOPE measures + MSCEIT only | 14 | -.07 | [-.14, .001] | .052 | 32.80% | 19.35 | .113 |
| Total positive symptoms | 19 | -.12 | [-.19, -.05] | .002 | 37.50% | 28.79 | .051 |
| Reality distortion symptoms | 13 | -.09 | [-.13, -.05] | < .001 | 0.00% | 5.57 | .936 |
| **EP - Negative symptoms** |  |  |  |  |  |  |  |
| **Primary Analysis** | 32 | -.18 | [-.22, -.13] | < .001 | 24.62% | 41.12 | .106 |
| SCOPE measures + MSCEIT only | 14 | -.19 | [-.25, -.14] | < .001 | 13.50% | 15.03 | .305 |
| **EP - Depressive symptoms** |  |  |  |  |  |  |  |
| **Primary Analysis** | 18 | .03 | [-.03, .08] | .329 | 11.93% | 19.30 | .311 |
| SCOPE measures + MSCEIT only | 10 | .02 | [-.08, .12] | .617 | 41.00% | 15.26 | .084 |
| Established Psychosis - Depression scale scores only | 13 | .03 | [-.05, .11] | .454 | 36.00% | 18.74 | .095 |
| Established Psychosis - PANSS Depression Factor scores only | 5 | .02 | [-.04, .08] | .365 | 0.00% | 0.56 | .968 |
| **EP – Disorganization symptoms** |  |  |  |  |  |  |  |
| **Primary Analysis** | 12 | -.21 | [-.24, -.19] | < .001 | 0.00% | 2.83 | .993 |
| SCOPE measures + MSCEIT only | 6 | -.16 | [-.22, -.10] | .001 | 0.00% | 3.04 | .694 |
| **Social functioning – Positive symptoms** |  |  |  |  |  |  |  |
| **Primary analysis** | 32 | -.23 | [-.28, -.17] | < .001 | 38.68% | 50.56 | .015 |
| Total positive symptoms | 19 | -.26 | [-.33, -.19] | < .001 | 33.20% | 26.95 | .080 |
| Reality distortion symptoms | 13 | -.17 | [-.25, -.07] | .002 | 45.10% | 21.88 | .039 |
| GAF removed | 27 | -.22 | [-.27, -.16] | < .001 | 40.70% | 43.84 | .016 |
| **Social functioning – Negative symptoms** |  |  |  |  |  |  |  |
| **Primary analysis** | 32 | -.39 | [-.47, -.31] | < .001 | 80.08% | 155.62 | < .001 |
| Outliers removed | 25 | -.38 | [-.43, -.32] | < .001 | 44.80% | 43.48 | .009 |
| GAF removed | 28 | -.39 | [-.47, -.30] | < .001 | 77.40% | 119.39 | < .001 |
|  |  |  |  |  |  |  |  |
|  |  |  |  |  |  |  |  |
| **Social functioning – Depressive symptoms** |  |  |  |  |  |  |  |
| **Primary analysis** | 18 | -.20 | [-.30, -.09] | .001 | 72.77% | 62.44 | < .001 |
| Outliers removed | 16 | -.20 | [-.29, -.10] | < .001 | 63.00% | 40.54 | .000 |
| Depression scale scores only | 13 | -.20 | [-.32, -.08] | .004 | 72.90% | 44.36 | < .001 |
| PANSS depression factor scores only | 5 | -.19 | [-.52, .18] | .225 | 77.90% | 18.07 | .001 |
| GAF removed | 17 | -.19 | [-.31, -.08] | .003 | 73.10% | 59.49 | < .001 |
| **Social functioning – Disorganization symptoms** |  |  |  |  |  |  |  |
| **Primary analysis** | 12 | -.32 | [-.43, -.20] | < .001 | 61.15% | 28.31 | .003 |
| GAF removed | 11 | -.31 | [-.43, -.18] | .000 | 63.90% | 27.71 | .002 |
| *Note:* EP, emotion processing; SCOPE: Social Cognition Psychometric Evaluation; MSCEIT: Mayer-Salovey-Caruso Emotional Intelligence Test; GAF: Global Assessment of Functioning; PANSS: Positive and Negative Syndrome Scale | | | | | | | |

**References**

1. Page MJ, McKenzie JE, Bossuyt PM, et al. The PRISMA 2020 statement: an updated guideline for reporting systematic reviews. *BMJ*. 2021;372:n71.

2. Moola S, Munn Z, Sears K, et al. Conducting systematic reviews of association (etiology): the Joanna Briggs Institute's approach. *JBI Evidence Implementation*. 2015;13(3):163-169.

3. Smith MJ, Horan WP, Cobia DJ, et al. Performance-based empathy mediates the influence of working memory on social competence in Schizophrenia. *Schizophrenia Bulletin*. 2014;40(4):824-834.

4. Abram SV, Karpouzian TM, Reilly JL, Derntl B, Habel U, Smith MJ. Accurate perception of negative emotions predicts functional capacity in schizophrenia. *Psychiatry Research*. 2014;216(1):6-11.

5. Martinez-Dominguez S, Penades R, Segura B, Gonzalez-Rodriguez A, Catalan R. Influence of social cognition on daily functioning in schizophrenia: Study of incremental validity and mediational effects. *Psychiatry Research*. 2015;225(3):374-380.

6. Green MF, Hellemann G, Horan WP, Lee J, Wynn JK. From perception to functional outcome in schizophrenia: Modeling the role of ability and motivation. *Archives of General Psychiatry*. 2012;69(12):1216-1224.

7. Kerr SL, Neale JM. Emotion Perception in Schizophrenia : Specific Deficit or Further Evidence of Generalized Poor Performance? *Journal of Abnormal Psychology*. 1993;102(2):312 – 318.

8. Bell M, Bryson G, Lysaker P. Positive and negative affect recognition in schizophrenia: a comparison with substance abuse and normal control subjects. *Psychiatry Research*. 1997/11/14/ 1997;73(1):73-82.

9. Kohler CG, Turner TH, Bilker WB, et al. Facial Emotion Recognition in Schizophrenia: Intensity Effects and Error Pattern. *American Journal of Psychiatry*. 2003/10/01 2003;160(10):1768-1774.

10. Heberlein AS, Adolphs R, Tranel D, Damasio H. Cortical Regions for Judgments of Emotions and Personality Traits from Point-light Walkers. *Journal of Cognitive Neuroscience*. 2004;16(7):1143-1158.

11. Young A, Perrett D, Calder A, et al. Facial Expressions of Emotion--Stimuli and Tests. The seventeenth mental measurements yearbook. Oxford: Pearson Assessment; 2002.

12. Hajduk M, Klein HS, Bass EL, Springfield CR, Pinkham AE. Implicit and explicit processing of bodily emotions in schizophrenia. *Cognitive Neuropsychiatry*. 2020;25(2):139-153.

13. Sahakian BJ, Owen AM. Computerized assessment in neuropsychiatry using CANTAB: discussion paper. *J R Soc Med*. Jul 1992;85(7):399-402.

14. Huang CL-C, Hsiao S, Hwu H-G, Howng S-L. The Chinese Facial Emotion Recognition Database (CFERD): A computer-generated 3-D paradigm to measure the recognition of facial emotional expressions at different intensities. *Psychiatry Research*. 2012/12/30/ 2012;200(2):928-932.

15. Wang K, Hoosain R, Lee TMC, Meng Y, Fu J, Yang R. Perception of Six Basic Emotional Facial Expressions by the Chinese. *Journal of Cross-Cultural Psychology*. 2006;37(6):623-629.

16. Hajduk M, Krajcovicova D, Zimanyiova M, Korinkova V, Heretik A, Pecenak J. Theory of mind - not emotion recognition - mediates the relationship between executive functions and social functioning in patients with schizophrenia. *Psychiatria Danubina*. 2018;30(3):292-298.

17. Charernboon T. Validity and reliability of the Thai version of the Faces Test. *J Med Assoc Thai*. 2017;100(6):42–45.

18. Derntl B, Finkelmeyer A, Toygar TK, et al. Generalized deficit in all core components of empathy in schizophrenia. *Schizophrenia Research*. 2009;108(1-3):197-206.

19. Brittain PJ, ffytche DH, Surguladze SA. Emotion perception and functional outcome in schizophrenia: The importance of negative valence and fear. *Psychiatry Research*. 2012;200(2-3):208-213.

20. Bölte S, Feineis-Matthews S, Leber S, Dierks T, Hubl D, Poustka F. The development and evaluation of a computer-based program to test and to teach the recognition of facial affect. *International Journal of Circumpolar Health*. 2002/09/01 2002;61(sup2):61-68.

21. Ekman P. *Pictures of facial affect.* Consulting Psychologists Press; 1976.

22. Erwin RJ, Gur RC, Gur RE, Skolnick B, Mawhinney-Hee M, Smailis J. Facial emotion discrimination: I. Task construction and behavioral findings in normal subjects. *Psychiatry Research*. 1992/06/01/ 1992;42(3):231-240.

23. Pijnenborg GHM, Withaar FK, van den Bosch RJ, Brouwer WH. Impaired Perception of Negative Emotional Prosody in Schizophrenia. *The Clinical Neuropsychologist*. 2007/08/20 2007;21(5):762-775.

24. Behere RV, Raghunandan VNGP, Venkatasubramanian G, Subbakrishna DK, Jayakumar PN, Gangadhar BN. TRENDS - A Tool for Recognition of Emotions in Neuropsychiatric DisorderS. *Indian Journal of Psychological Medicine*. 2008;30(1):32-38.

25. Mayer JD, Salovey P, Caruso DR, Sitarenios G. Measuring emotional intelligence with the MSCEIT V2.0. *Emotion*. Mar 2003;3(1):97-105.

26. Bagby RM, Taylor GJ, Ryan D. Toronto Alexithymia Scale: Relationship with Personality and Psychopathology Measures. *Psychotherapy and Psychosomatics*. 1986;45(4):207-215.

27. Salovey P, Mayer JD, Goldman SL, Turvey C, Palfai TP. Emotional attention, clarity, and repair: Exploring emotional intelligence using the Trait Meta-Mood Scale. In: Pennebaker JW, ed. *Emotion, Disclosure, and Health*. American Psychological Association; 1995.

28. Kay SR, Fiszbein A, Opler LA. The Positive and Negative Syndrome Scale (PANSS) for Schizophrenia. *Schizophrenia Bulletin*. 1987;13(2):261-276.

29. Andreasen NC, Arndt S, Alliger R, Miller D, Flaum M. Symptoms of Schizophrenia: Methods, Meanings, and Mechanisms. *Archives of General Psychiatry*. 1995;52(5):341-351.

30. Overall JE, Gorham DR. The Brief Psychiatric Rating Scale. *Psychological Reports*. 1962;10(3):799-812.

31. Yung AR, Yung AR, Pan Yuen H, et al. Mapping the Onset of Psychosis: The Comprehensive Assessment of At-Risk Mental States. *Australian & New Zealand Journal of Psychiatry*. 2005;39(11-12):964-971.

32. Haro JM, Kamath SA, Ochoa S, et al. The Clinical Global Impression–Schizophrenia scale: a simple instrument to measure the diversity of symptoms present in schizophrenia. *Acta Psychiatrica Scandinavica*. 2003;107(s416):16-23.

33. Mucci A, Galderisi S, Merlotti E, et al. The Brief Negative Symptom Scale (BNSS): Independent validation in a large sample of Italian patients with schizophrenia. *European Psychiatry*. 2015;30(5):641-647.

34. Kring AM, Gur RE, Blanchard JJ, Horan WP, Reise SP. The Clinical Assessment Interview for Negative Symptoms (CAINS): Final Development and Validation. *American Journal of Psychiatry*. 2013/02/01 2013;170(2):165-172.

35. Addington D, Addington J, Maticka-tyndale E. Assessing Depression in Schizophrenia: The Calgary Depression Scale. *The British Journal of Psychiatry*. Dec 1993

2024-03-26 1993;163(S22):39-44.

36. Beck AT, Steer RA, Brown GK. *The Beck Depression Inventory—Second Edition.* . The Psychological Corporation; 1996.

37. Hamilton M. A RATING SCALE FOR DEPRESSION. *Journal of Neurology, Neurosurgery &amp;amp; Psychiatry*. 1960;23(1):56.

38. Birchwood M, Smith J, Cochrane R, Wetton S, Copestake S. The Social Functioning Scale the Development and Validation of a New Scale of Social Adjustment for use in Family Intervention Programmes with Schizophrenic Patients. *British Journal of Psychiatry*. 1990;157(6):853-859.

39. Morosini PL, Magliano L, Brambilla L, Ugolini S, Pioli R. Development, reliability and acceptability of a new version of the DSM-IV Social and Occupational Functioning Assessment Scale (SOFAS) to assess routine social functioning. *Acta Psychiatr Scand*. Apr 2000;101(4):323-9.

40. Association AP. *Diagnostic and statistical manual of mental disorders - 4th Edition*. APA; 1994.

41. Schneider LC, Struening EL. SLOF: a behavioral rating scale for assessing the mentally ill. *Social Work Research and Abstracts*. 1983;19(3):9-21.

42. Heinrichs DW, Hanlon TE, Carpenter WT, Jr. The Quality of Life Scale: An Instrument for Rating the Schizophrenic Deficit Syndrome. *Schizophrenia Bulletin*. 1984;10(3):388-398.

43. Weissman MM. *Social adjustment scale-self report (SAS-SR): User's manual*. Multi-Health Systems Incorporated; 1999.

44. Goodman SH, Sewell DR, Cooley EL, Leavitt N. Assessing levels of adaptive functioning: The Role Functioning Scale. *Community Mental Health Journal*. 1993/04/01 1993;29(2):119-131.

45. Wiersma D, DeJong A, Ormel J. The Groningen Social Disabilities Schedule. *International Journal of Rehabilitation Research*. 1988;11(3):213-224.

46. Wallace CJ. Community and Interpersonal Functioning in the Course of Schizophrenic Disorders. *Schizophrenia Bulletin*. 1984;10(2):233-257.

47. Schooler NH, Weissman G, Hogarty G, Hargreaves WA, Attkisson CC, Sorenson J. *Resource materials for community mental health program evaluators*. National Institute of Mental Health; 1979.

48. Rybarczyk B. Social and occupational functioning assessment scale (SOFAS). In: Kreutzer J, DeLuca J, Caplan B, editors, eds. *Encyclopedia of clinical neuropsychology*. Springer Reference; 2011:2313.

49. Organization WH. Measuring Health and Disability : Manual for WHO Disability Assessment Schedule (WHODAS 2.0). World Health Organization; 2010.

50. Harrer M, Cuijpers P, Furukawa TA, Ebert DD. *Doing Meta-Analysis With R: A Hands-On Guide*. 1st ed. Chapman & Hall/CRC Press; 2021.

51. Baujat B, Mahé C, Pignon JP, Hill C. A graphical method for exploring heterogeneity in meta-analyses: Application to a meta-analysis of 65 trials. *Statistics in Medicine*. 2002;21(18):2641-2652.

52. Ventura J, Wood RC, Hellemann GS. Symptom domains and neurocognitive functioning can help differentiate social cognitive processes in schizophrenia: a meta-analysis. *Schizophr Bull*. Jan 2013;39(1):102-11.

53. Addington D, Addington J, Maticka-tyndale E. Assessing Depression in Schizophrenia: The Calgary Depression Scale. *British Journal of Psychiatry*. 1993;163(S22):39-44.

54. Wallwork RS, Fortgang R, Hashimoto R, Weinberger DR, Dickinson D. Searching for a consensus five-factor model of the Positive and Negative Syndrome Scale for schizophrenia. *Schizophrenia Research*. 2012/05/01/ 2012;137(1):246-250.
